# Activation of IL-27 signalling promotes development of postinfluenza pneumococcal pneumonia

**DOI:** 10.1002/emmm.201302890

**Published:** 2013-10-29

**Authors:** Ju Cao, Dongsheng Wang, Fang Xu, Yi Gong, Hong Wang, Zixin Song, Dageng Li, Hua Zhang, Dairong Li, Liping Zhang, Yun Xia, Huajian Xu, Xaiofei Lai, Shihui Lin, Xuemei Zhang, Guosheng Ren, Yubing Dai, Yibing Yin

**Affiliations:** 1 Department of Laboratory Medicine, The First Affiliated Hospital of Chongqing Medical UniversityChongqing, China; 2 National Laboratory of Medical Molecular Biology, Institute of Basic Medical Sciences, Chinese Academy of Medical Sciences & Peking Union Medical CollegeBeijing, China; 3 Department of Laboratory Medicine, Affiliated Hospital of North Sichuan Medical CollegeNanchong, China; 4 Department of Emergency and Intensive Care Unit, The First Affiliated Hospital of Chongqing Medical UniversityChongqing, China; 5 Department of Blood Transfusion, The First Affiliated Hospital of Chongqing Medical UniversityChongqing, China; 6 Key Laboratory of Diagnostic Medicine designated by the Ministry of Education, Chongqing Medical UniversityChongqing, China; 7 Department of Obstetrics and Gynecology, The First Affiliated Hospital of Chongqing Medical UniversityChongqing, China; 8 Department of Respiratory Disease, The First Affiliated Hospital of Chongqing Medical UniversityChongqing, China; 9 Molecular Oncology and Epigenetics Laboratory, The First Affiliated Hospital of Chongqing Medical UniversityChongqing, China; 10 Center for Nuclear Receptors and Cell Signaling, University of HoustonHouston, Texas, USA

**Keywords:** influenza virus, IL-17, IL-27, *Streptococcus pneumoniae*, γδ T cells

## Abstract

Postinfluenza pneumococcal pneumonia is a common cause of death in humans. However, the role of IL-27 in the pathogenesis of secondary pneumococcal pneumonia after influenza is unknown. We now report that influenza infection induced pulmonary IL-27 production in a type I IFN-α/β receptor (IFNAR) signalling-dependent manner, which sensitized mice to secondary pneumococcal infection downstream of IFNAR pathway. Mice deficient in IL-27 receptor were resistant to secondary pneumococcal infection and generated more IL-17A-producing γδ T cells but not αβ T cells, thereby leading to enhanced neutrophil response during the early phase of host defence. IL-27 treatment could suppress the development of IL-17A-producing γδ T cells activated by *Streptococcus pneumoniae* and dendritic cells. This suppressive activity of IL-27 on γδ T cells was dependent on transcription factor STAT1. Finally, neutralization of IL-27 or administration of IL-17A restored the role of γδ T cells in combating secondary pneumococcal infection. Our study defines what we believe to be a novel role of IL-27 in impairing host innate immunity against pneumococcal infection.

## Introduction

Influenza has a substantial impact on global health, and it accounts for significant fatalities annually worldwide (Nair *et al*, 2011). Clinical data have shown that most deaths following influenza virus infection are attributed to the complications of secondary bacterial pneumonia, mainly caused by *Streptococcus pneumoniae* (Brundage 2006; CDC, 2009; van der Sluijs *et al*, 2004). Considering the risk of influenza pandemics and the increasing prevalence of pneumococcal antibiotic resistance, it is important to elucidate potential mechanisms involved in the pathogenesis of postinfluenza pneumococcal pneumonia.

Complex interactions among viral, pneumococcal and host factors contribute to the associations between primary viral and secondary pneumococcal infection. Earlier reports have proposed that disruption of airway epithelial layer, induction of inhibitory cytokine IL-10 and increased pneumococcal adherence due to up-regulation of platelet-activating factor receptor were implicated in promoting postinfluenza secondary pneumococcal pneumonia (Cao *et al*, 2009; McCullers & Rehg 2002; van der Sluijs *et al*, 2004). Recently, suppression of neutrophil activity by type I IFNs and impairment of alveolar macrophage function by IFN-γ were also found to contribute to this process (Kudva *et al*, 2011; Li *et al*, 2012; Nakamura *et al*, 2011; Shahangian *et al*, 2009; Sun & Metzger 2008). These data suggest that host innate system undergoes some education following primary viral infection that alters the way it responds to secondary pneumococcal infection.

IL-27 is a heterodimeric cytokine composed of the Epstein–Barr virus-induced gene 3 (EBI3) and IL-27p28, which signals through a receptor complex composed of T-cell cytokine receptor (TCCR)/WSX-1 and gp130 (Hunter & Kastelein 2012; Villarino *et al*, 2003). Initial studies have demonstrated that IL-27 contributed to Th1 immunity at the early step of differentiation (Kamiya *et al*, 2004; Lucas *et al*, 2003). However, subsequent studies have shown that IL-27 could suppress Th1, Th2 and Th17 cell responses in a context-dependent manner (Stumhofer *et al*, 2006; Villarino *et al*, 2005; Yoshimoto *et al*, 2007). While the regulatory effects of IL-27 on adaptive immunity have been well established, their role in regulating innate immunity, especially during bacterial infection is still largely unknown. In this study, we have developed a murine model of postinfluenza pneumococcal pneumonia using mice defective for IL-27 receptor signalling (IL-27R^−/−^ mice) to investigate the effects of IL-27 induced during influenza infection on host immunity to pneumococcal infection. For the first time, we found that IL-27 exerted regulatory effects during postinfluenza pneumococcal pneumonia. The strikingly elevated pulmonary IL-27 induced by influenza infection via type I IFN-α/β receptor (IFNAR) signalling pathway predisposed to secondary pneumococcal pneumonia, which was attributable to IL-27-mediated suppression of innate IL-17A production in γδ T cells but not the adaptive Th17-cell response in a STAT1-dependent way, thereby leading to depressed neutrophil response. Our data point to a mechanism by which IL-27 has a deleterious effect on the clearance and survival following secondary pneumococcal infection after influenza infection.

## Results

### Efficient clearance of *S. pneumoniae* from the lung requires IL-17A

**Figure 1:**
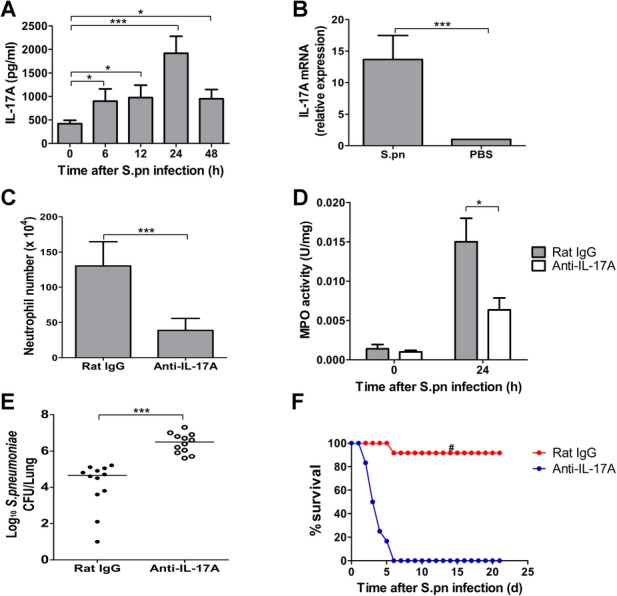
IL-17A was required for efficient clearance of S. *pneumoniae* in the lung. A  Dynamic changes of lung IL-17A levels in the mice upon intranasal challenge with Type 3 *S. pneumoniae* (*n* = 5). B  Lung IL-17A mRNA levels at 24 h after pneumococcal challenge (*n* = 5). C  Lung neutrophil numbers at 24 h after inhibition of IL-17A. Either anti-IL-17A neutralizing antibodies or control IgG were injected i.p. in mice, and mice were subsequently infected with *S. pneumoniae* intranasally (*n* = 5). D  Lung MPO activity at 24 h after inhibition of IL-17A (*n* = 5). E  Pulmonary pneumococcal burdens at 48 h after inhibition of IL-17A. The horizontal lines indicate the median CFU per lung (*n* = 12). F  Survival was examined for 21 days after pneumococcal challenge in the presence or absence of anti-IL-17A neutralizing antibodies (*n* = 12). *p < 0.05, ***p < 0.001 when compared between groups denoted by horizontal lines; ^#^*p* < 0.05 when compared with mice treated with anti-IL-17A.

IL-17A is a critical cytokine for neutrophil accumulation and activity (Hoshino *et al*, 1999; Wong *et al*, 2010). To investigate the role of IL-17A in the accumulation of neutrophils in the lung, we measured IL-17A levels during pneumococcal infection established with Type 3 *S. pneumoniae*. IL-17A in the lungs significantly increased and peaked at 24 h after *S. pneumoniae* challenge (Fig 1A). Importantly, the mRNA levels of IL-17A in pneumococci-challenged lungs were much higher than those in control lungs (Fig 1B). To understand the effect of IL-17A on neutrophil accumulation in the lung, a neutralizing antibody against IL-17A was used to inhibit the function of IL-17A. The number of lung neutrophils in the mice treated with anti-IL-17A antibodies was significantly reduced relative to mice treated with isotypical antibodies (Fig 1C). MPO activity, a marker of neutrophil function, was also significantly less in homogenized lungs of mice treated with anti-IL-17 antibodies (Fig 1D). Besides, IL-17A neutralization resulted in significantly increased pneumococcal burdens in the lungs (Fig 1E), and the survival rate of mice treated with anti-IL-17A antibodies was significantly lower than that of control mice (Fig 1F). These data suggest that IL-17A was required for neutrophil response upon pneumococcal infection, which plays an important role in protecting against pneumococcal pneumonia.

### γδ T cells are the major producers of IL-17A during pneumococcal pneumonia

αβ T cells, NKT cells and γδ T cells have been reported to regulate inflammatory diseases in an IL-17A-dependent manner (Rendon & Choudhry^2012^; Sutton *et al*, 2012). To determine the phenotype of IL-17A-producing cells at the early stage of pneumococcal pneumonia, lung cells were isolated 24 h after pneumococcal infection, stained for cell surface markers and cytoplasmic IL-17A, and analyzed by flow cytometry (supplementary Fig 1A). The enhanced IL-17A production was predominately by γδ T cells with limited IL-17A production in other T cells (supplementary Fig 1B and C). Besides, IL-17A response in γδ T cells was both faster and stronger than that in Th17 cells (supplementary Fig 1D). These data suggest a crucial role of γδ T cells in contributing to lung IL-17A production during pneumococcal pneumonia.

To further elucidate how γδ T cells elicit protective immunity against *S. pneumoniae* lung infection, we compared IL-17A production and pneumococcal clearance in both γδ T-cell-deficient and wild-type (WT) mice. WT mice exhibited an early burst of IL-17A gene expression, which peaked at 8 h and declined by 24 h (supplementary Fig 1E). In contrast, γδ T-cell-deficient mice lacked this early induction of IL-17A, while both IL-22 and IL-21 were induced in γδ T-cell-deficient and WT mice. ELISA assays further confirmed an impaired IL-17A protein production but not IL-22 and IL-21 in the lungs of γδ T-cell-deficient mice (supplementary Fig 1F). The lungs from infected mice were also examined microscopically. WT mice had the infiltration of large amounts of inflammatory cells, especially neutrophils, while γδ T-cell-deficient mice had less neutrophil infiltration (supplementary Fig 1G and H). There was also significantly less MPO activity in homogenized lungs of γδ T-cell-deficient mice compared with WT mice (supplementary Fig 1I). At 48 h following *S. pneumoniae*infection, lungs from WT mice contained significantly reduced median bacterial loads as compared with γδ T-cell-deficient mice (supplementary Fig 1J). And a significantly higher mortality was observed in γδ T-cell-deficient mice when compared with WT mice (supplementary Fig 1K). Additionally, treatment of γδ T-cell-deficient mice with exogenous recombinant IL-17A could enhance lung neutrophil recruitment (supplementary Fig 2A) and up-regulate MPO activity in lung homogenates (supplementary Fig 2B). IL-17A treatment could also significantly decrease lung pneumococcal burdens (supplementary Fig 2C) and improve survival times in γδ T-cell-deficient mice (supplementary Fig 2D). Together, these data suggest that IL-17A in γδ T cells plays a key role in mediating neutrophil response against *S. pneumoniae* infection.

### Influenza virus inhibits IL-17A production by γδ T cells upon secondary pneumococcal infection

**Figure 2:**
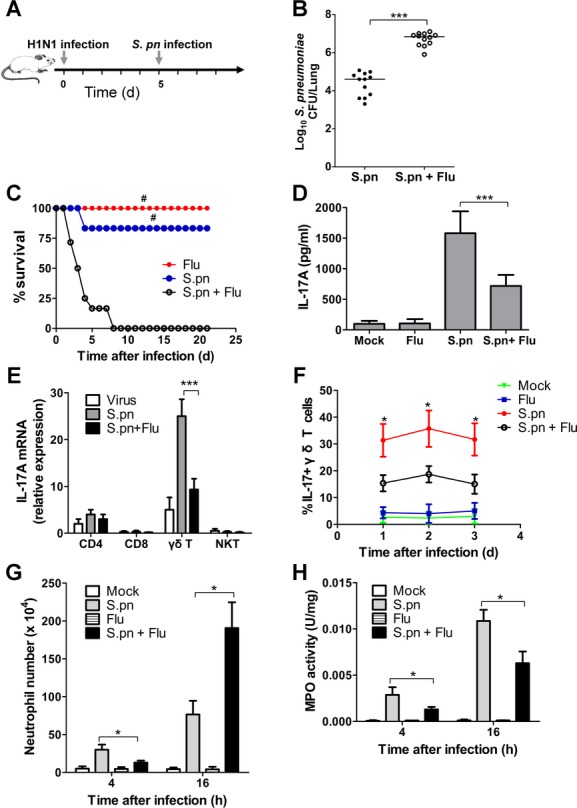
Influenza virus inhibited IL-17A production by γ´ T cells in postinfluenza pneumonia. A  Schematic representation model of postinfluenza pneumonia in C57BL/6 mice. B  Pulmonary pneumococcal burdens in the mice at 48 h after primary or secondary pneumococcal infection (*n* = 12). C  Survival for groups of mice after influenza infection, *S. pneumoniae* infection or secondary pneumococcal infection following influenza infection (*n* = 12). D  Lung IL-17A levels in the indicated groups of mice at 24 h after pneumococcal infection (*n* = 5). E  γδ T cells, CD4+ T cells, CD8+ T cells and NKT cells were purified by cell sorting. IL-17A gene expression was measured from different cell types. Results are presented relative to GAPDH (*n* = 5). F  Percentages of IL-17A producers among γδ T cells in the lungs of groups of mice at indicated times following pneumococcal challenge (*n* = 5). G  Lung neutrophil numbers in the mice at 4, 16 h following pneumococcal challenge (*n* = 5). H  Lung MPO activity in the mice at 4, 16 h following pneumococcal challenge (*n* = 5). *p < 0.05, ***p < 0.001 when compared between mice infected with *S. pneumoniae* alone and mice infected with influenza virus plus *S. pneumoniae*; ^#^*p* < 0.05 when compared with mice of secondary pneumococcal infection.

Since IL-17A in γδ T cells was shown to be critical for host defence against *S. pneumoniae*, and influenza virus enhanced sensitivity of mice to secondary *S. pneumoniae* infection (Shahangian *et al*, 2009), we proposed that influenza infection may inhibit IL-17A production by γδ T cells in the lung. Clinically most secondary pneumococcal infection develops within the first 2 weeks after primary influenza infection, and secondary pneumococcal infection is most lethal between 5 and 7 days following influenza infection (McCullers & Rehg 2002; McNamee & Harmsen^2006^). Thus, we established an infection model in which mice were challenged intranasally with *S. pneumoniae* at day 5 after influenza infection (Fig 2A). Similar to prior reports, in mice with prior influenza infection, a markedly increase in pulmonary pneumococcal burden was detected at 48 h after secondary *S. pneumoniae* infection (Fig 2B), and a significantly higher mortality was observed in virus/*S. pneumoniae*-infected mice when compared with *S. pneumoniae*-infected or virus-infected mice (Fig 2C).

In order to identify whether there was some dysregulated production of IL-17A during secondary pneumococcal pneumonia, cytokine/chemokine/growth factor was measured in lung homogenates of mice infected with virus alone, *S. pneumoniae* alone or virus plus *S. pneumoniae*. The results showed that by 24 h after pneumococcal infection there was a significant increase of protein production of CXCL1, IL-6, TNF-α, CXCL10 and G-CSF in virus/*S. pneumoniae*-infected mice relative to mice given virus or *S. pneumoniae* alone (supplementary Fig 3), which is likely due to the markedly higher lung pneumococcal burdens in virus/*S. pneumoniae*-infected mice. However, IL-17A secretion was significantly reduced in virus/*S. pneumoniae*-infected mice compared with mice given *S. pneumoniae* alone (Fig 2D), suggesting that primary influenza infection appeared to lead to a selective attenuation of IL-17A. We further characterized the source of IL-17A by isolating different cell population from mice lungs. The isolated γδ T cells from mice infected with *S. pneumoniae* showed the highest level of IL-17A gene expression, but its level was significantly reduced in mice infected with virus/*S. pneumoniae* (Fig 2E). Besides, the percentage of IL-17A-producing γδ T cells was significantly reduced at different time points in mice infected with virus/*S. pneumoniae* relative to mice infected with *S. pneumoniae* alone (Fig 2F).

In addition, neutrophil recruitment and activity in the lungs were studied in this model. Since marked differences in lung pneumococcal burdens were noted 48 h after pneumococcal infection, which might impact neutrophil levels, we examined neutrophil recruitment and MPO activity at earlier time point of 4 h after infection, before differences in pneumococcal burdens became apparent (data not shown). Mice infected with virus/*S. pneumoniae* had slightly but significantly reduced levels of neutrophil recruitment (Fig 2G) and MPO activity relative to mice infected with *S. pneumoniae* alone (Fig 2H). By 16 h following infection, although neutrophil numbers were significantly higher after secondary pneumococcal pneumonia (Fig 2G), MPO activity in virus/*S. pneumoniae*-infected mice was significantly lower than that in *S. pneumoniae*-infected mice (Fig 2H). These data suggest that influenza infection impaired neutrophil response against pneumococcal infection by negatively regulating IL-17A production in γδ T cells.

### Influenza induces the production of anti-inflammatory cytokine IL-27 in an IFNAR-dependent manner

**Figure 3:**
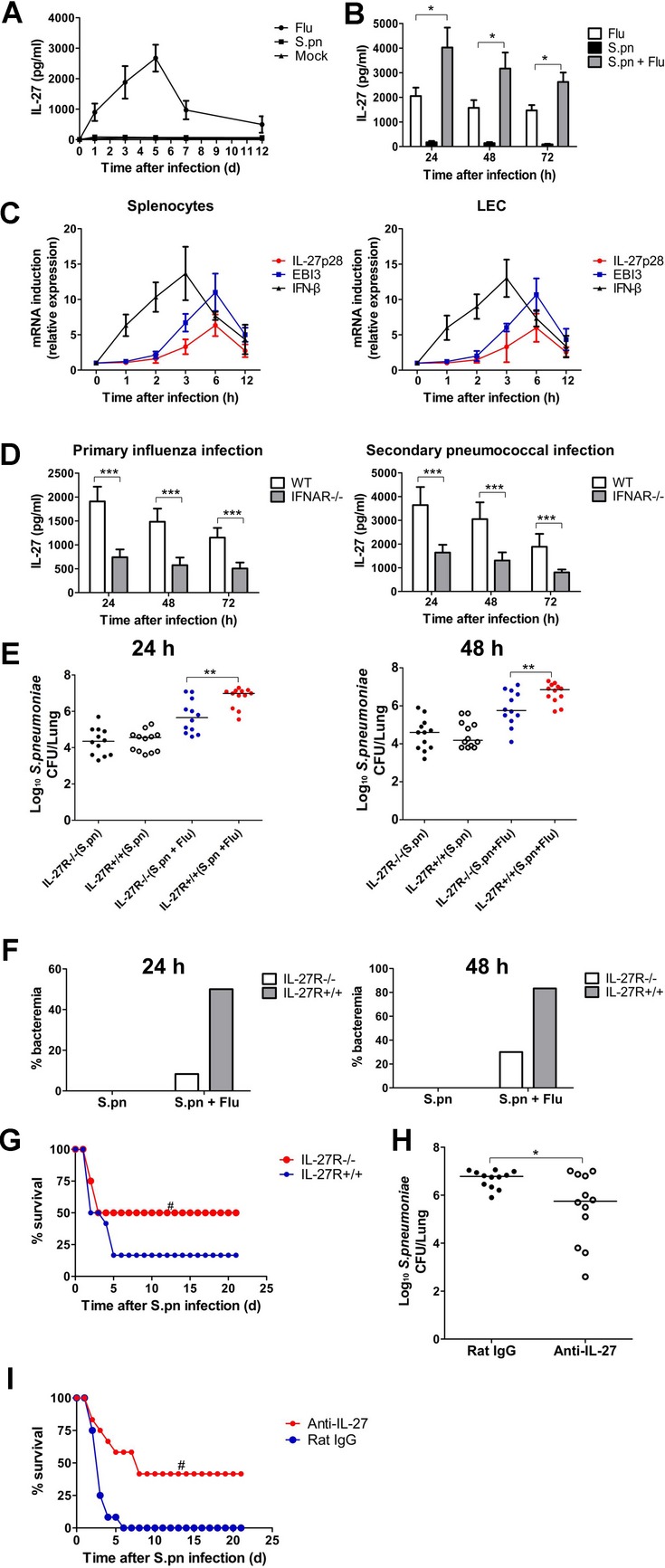
Influenza-infected IL-27R-deficient mice were resistant to secondary pneumococcal pneumonia. A  Lungs from indicated groups of mice were harvested at the designated time points for assessment of IL-27 by ELISA (*n* = 5). B  Lung IL-27 production in the groups of mice at 24, 48, 72 h after influenza infection, *S. pneumoniae* infection or secondary pneumococcal infection following influenza infection (*n* = 5). C  WT splenocytes or LEC were stimulated with influenza virus (MOI = 1) for the indicated time points. IFN-β, EBI3, and IL-27p28 gene transcript level was detected by quantitative PCR (*n* = 3). D  IL-27 production in the lungs of IFNAR-deficient or WT mice after primary influenza infection or secondary pneumococcal infection (*n* = 5). E  Pulmonary pneumococcal burdens in IL-27R-deficient and WT mice at 24 or 48 h following primary pneumococcal infection or secondary pneumococcal infection (*n* = 12). F  Incidence of bacteraemia was measured in IL-27R-deficient and WT mice at 24 or 48 h following primary pneumococcal infection or secondary pneumococcal infection (*n* = 12). G  Survival for indicated groups of mice following secondary pneumococcal infection (*n* = 12). H  Pulmonary pneumococcal burdens in WT mice treated with anti-IL-27 blocking antibodies at 48 h after secondary pneumococcal infection (*n* = 12). I  Survival for indicated groups of mice treated with anti-IL-27 neutralizing antibodies or isotypical IgG after secondary pneumococcal infection (*n* = 12). *p < 0.05, **p < 0.01, ***p < 0.001 when compared between groups denoted by horizontal lines; ^#^*p* < 0.05 when compared between indicated groups of mice.

To determine the mechanism by which influenza promoted mice to secondary pneumococcal pneumonia, we analyzed the kinetics of the immune response to influenza infection. Since IL-27 is an important regulatory cytokine that can limit ongoing immune responses depending on context (Hunter & Kastelein 2012), we first examined the induction of IL-27 *in vivo*, and found that influenza infection elicited elevated levels of IL-27 and IL-27 peaked on day 5 and persisted to day 12 (Fig 3A), which correlated with the timing of high susceptibility to secondary *S. pneumoniae* infection on 5–7 days after primary influenza infection. Following *S. pneumoniae* challenge on day 5 after primary influenza infection, we also found that secondary infected mice had a synergistic increase in IL-27 production compared with mice infected with either *S. pneumoniae* or influenza virus alone (Fig 3B). To determine the primary cell source from which IL-27 was induced *in vivo*, murine bone-marrow-derived dendritic cells (BMDC), monocytes, lung epithelial cells (LEC) and lymphocytes were isolated, and influenza virus infection resulted in a significant IL-27 production in murine BMDC, monocytes, LEC and lymphocytes *in vitro* (supplementary Fig 4A). Although the production of IL-27 induced by heat-killed *S. pneumoniae* (HkSp) was limited, we did find significant enhancing effects of HkSp on IL-27 secretion induced by influenza virus. This was true for all cells from C57BL/6 mice, indicating that influenza virus was a potent stimulus for IL-27 secretion.

Recently, it has been demonstrated that type I IFNs mediated IL-27 gene expression (Remoli *et al*, 2007). To further investigate if influenza-induced IL-27 is regulated by type I IFNs, we firstly studied the relative kinetics of IFN-β *versus* IL-27 transcript expression (Fig 3C). Detectable levels of IFN-β appeared 1 h after influenza infection and peak at ∼3 h, while induction of IL-27p28 or EBI3 appeared delayed relative to IFN-β, with significant mRNA detection occurring ∼3 h after influenza infection and peaking at ∼6 h. Remarkably, IL-27 production was significantly reduced in IFNAR-deficient BMDC, monocytes, LEC and lymphocytes after influenza infection with or without HkSp co-stimulation compared with WT cells (supplementary Fig 4B). *In vivo*, we further found that IFNAR-deficient mice after influenza infection had much less IL-27 protein in the lungs compared with WT mice (Fig 3D). All these data suggest that influenza infection up-regulated IL-27 production in an IFNAR-dependent manner.

### Influenza-induced IL-27 promotes secondary pneumococcal infection

The coincident timing between the appearance of IL-27 and enhanced susceptibility to secondary *S. pneumoniae* infection suggested this regulatory cytokine may contribute to the sensitivity to secondary pneumococcal pneumonia. To test this hypothesis, WT mice or mice lacking IL-27 receptor (IL-27R) were inoculated with *S. pneumoniae* 5 days after influenza infection. IL-27R-deficient mice did not have significant difference in lung viral burdens or in weight loss, when compared with WT mice after influenza infection (supplementary Fig 5). Besides, no significant difference was observed in lung pneumococcal burdens of WT and IL-27R-deficient mice following *S. pneumoniae* infection alone (Fig 3E). However, lungs from virus/*S. pneumoniae*-infected IL-27R-deficient mice contained about tenfold fewer median bacterial loads when compared with WT mice as early as 24 h, with significant differences persisting for up to 48 h (Fig 3E). Similarly, markedly lower rates of bacteraemia were noted in virus/*S. pneumoniae*-infected IL-27R-deficient mice when compared with WT mice (Fig 3F). These differences in pulmonary and systemic pneumococcal loads were associated with significantly decreased mortality in IL-27R-deficient mice (Fig 3G), and this correlation between pneumococcal burdens and mortality was expected during pneumococcal infection, which was consistent with the reports as described in our previous studies and others when studying the protection of novel pneumococcal protein vaccines against pneumococcal infection (Giefing *et al*, 2008; Gong *et al*, 2011; Min *et al*, 2012). Hence, the induction of IL-27 did not affect the viral or pneumococcal clearance in naive mice, but markedly increased susceptibility to secondary pneumococcal infection. Additionally, WT mice were treated with IL-27-neutralizing antibodies or mouse IgG and then infected as described above. Following influenza infection, mice treated with anti-IL-27 antibodies had significantly enhanced pulmonary clearance upon secondary pneumococcal challenge (Fig 3H) and significantly increased survival rates (Fig 3I) when compared with IgG-treated mice, which were consistent with those observed in IL-27R-deficent mice.

### IL-27 induced by influenza infection suppresses IL-17A production in γδ T cells upon secondary pneumococcal infection

**Figure 4:**
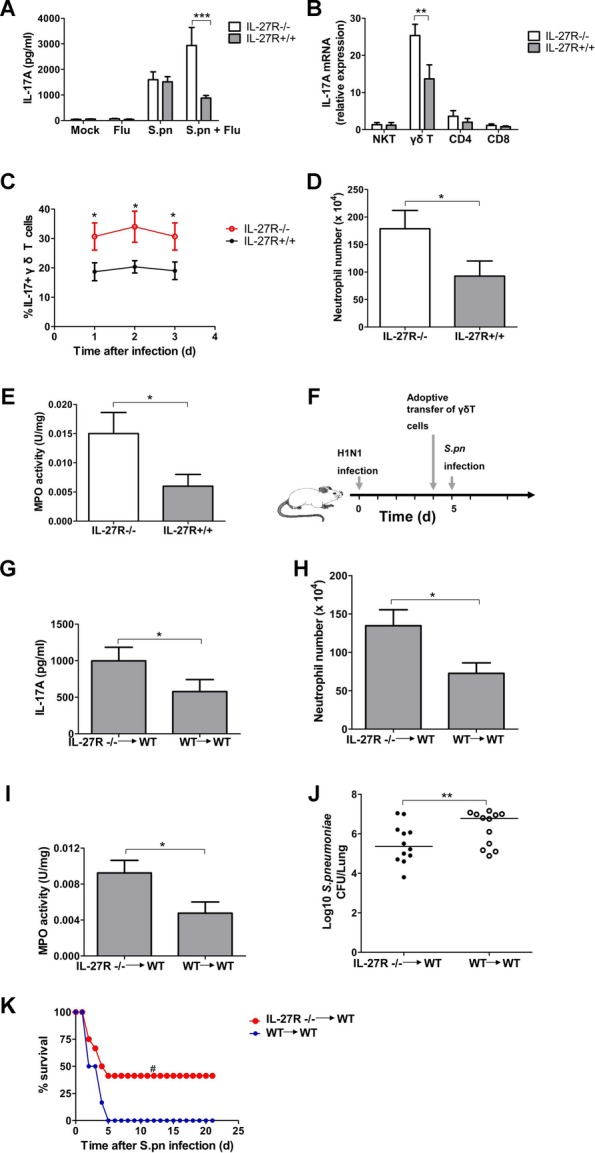
IL-27 negatively regulated IL-17A production by γ´ T cell upon pneumococcal infection in mice. A  IL-17A production in the lungs from IL-27R-deficient and WT mice at 24 h after secondary pneumococcal challenge following influenza infection (*n* = 5). B  γδ T cells, CD4+ T cells, CD8+ T cells and NKT cells from IL-27R-deficient and WT mice were purified by cell sorting at 24 h after secondary pneumococcal infection. IL-17A gene expression was measured from different cell types (*n* = 5). C  Percentages of IL-17A producers among γδ T cells in the lungs from IL-27R-deficient and WT mice at indicated times following secondary pneumococcal challenge (*n* = 5). D  Lung neutrophil numbers in IL-27R-deficient and WT mice at 24 h following secondary pneumococcal challenge (*n* = 5). E  Lung MPO activity in IL-27R-deficient and WT mice at 24 h following secondary pneumococcal challenge (*n* = 5). F  Schematic representation model of γδ T cells adoptive transfer experiment. G  Lung IL-17A levels were determined at 24 h after secondary pneumococcal infection (*n* = 5). H  Lung neutrophil numbers in WT mice transferred with IL-27R-deficient or WT γδ T cells at 24 h after secondary pneumococcal infection (*n* = 5). I  Lung MPO activity at 24 h after secondary pneumococcal infection (*n* = 5). J  Pulmonary pneumococcal burdens at 48 h after secondary pneumococcal infection (*n* = 12). K  Survival for WT mice transferred with IL-27R-deficient or WT γδ T cells after secondary pneumococcal infection (*n* = 12). *p < 0.05, **p < 0.01, ***p < 0.001 when compared between groups denoted by horizontal lines; ^#^*p* < 0.05 when compared with mice transferred with WT γδ T cells.

The findings that IL-27 sensitized mice to secondary pneumococcal pneumonia suggested this cytokine profoundly altered host anti-pneumococcal defence in the lung. Since a variety of cytokines, chemokines and growth factors were produced in the lungs during microbial infection, which contributed to recruit and activate inflammatory cells with concomitant clearance of microbes (Ward 2012), we next examined whether there were any differences in the levels of lung inflammatory mediators in influenza-infected WT and IL-27R-deficient mice upon secondary *S. pneumoniae* challenge. We focused on differences in this inflammatory response at earlier time points, and lung homogenates obtained from WT and IL-27R-deficient mice were analyzed at 24 h after secondary pneumococcal infection. Following influenza or *S. pneumoniae* infection alone, there was no significant difference in cytokine/chemokine/G-CSF production between WT and IL-27R-deficient mice (Fig 4A and supplementary Fig 6). In postinfluenza pneumococcal pneumonia, however, significantly enhanced IL-17A production was observed in IL-27R-deficient mice (Fig 4A), whereas the production of CXCL1, IL-6, TNF-α, CXCL10 and G-CSF was identical in both groups (supplementary Fig 6). Further identification of the specific cell population producing IL-17A demonstrated that lung γδ T cells from IL-27R-deficient mice had a significantly higher level of IL-17A gene expression compared with those from WT mice (Fig 4B), and the percentage of IL-17A-producing γδ T cells was also significantly higher at different time points in IL-27R-deficient mice (Fig 4C). Accordingly, IL-27R-deficient mice showed significantly increased neutrophil recruitment in their lungs relative to their WT counterparts (Fig 4D), and MPO activity was also significantly higher in IL-27R-deficient mice (Fig 4E). These results indicate that IL-27 could suppress IL-17A production in γδ T cells during secondary pneumococcal infection.

To investigate that the adoptive transfer of IL-27R-deficient γδ T cells that are nonresponsive to IL-27 should improve anti-pneumococcal defence in WT mice with postinfluenza pneumococcal infection, γδ T cells were purified from IL-27R-deficient or WT mice by cell sorting (supplementary Fig 7), and were transferred intratracheally (i.t.) into WT mice following primary influenza infection, and then mice were intranasally challenged with *S. pneumoniae* (Fig 4F). WT mice receiving IL-27R-deficient γδ T cells demonstrated significantly elevated IL-17A production (Fig 4G), and increased neutrophil recruitment (Fig 4H) as well as up-regulated MPO activity (Fig 4I) in their lungs. Besides, these mice had a significantly enhanced ability to clear *S. pneumoniae* (Fig 4J), and they were associated with a significantly decreased mortality (Fig 4K).

Having observed that IL-17A in γδ T cells was the significant cytokine for enhanced clearance of secondary pneumococcal pneumonia observed in IL-27R-deficient mice, neutralization of IL-17A would dramatically increase sensitivity of influenza-infected IL-27R-deficient mice. Therefore, we used neutralizing antibodies against IL-17A in 27R-deficient mice to solidify the role of IL-17A in mediating the effects of IL-27. IL-17A depletion resulted in a significant decrease of neutrophil recruitment (supplementary Fig 8A) and MPO activity (supplementary Fig 8B) in the lungs of IL-27R-deficient mice following secondary *S. pneumoniae* challenge. Besides, the pneumococcal counts in IL-17A-depleted IL-27R-deficient mice were significantly increased (supplementary Fig 8C), and a significantly enhanced morality was observed in these mice (supplementary Fig 8D), while isotypical IgG-treated IL-27R-deficient mice had similar parameters with WT mice. Interestingly, neutralization of IL-17A did not significantly change pulmonary pneumococcal burdens in WT mice (supplementary Fig 8C), indicating that IL-17A was produced at a functionally ineffective level in WT mice during secondary pneumococcal pneumonia. All together, these data support a mechanism by which influenza induced IL-27, which through inhibition of IL-17A production in γδ T cells attenuated neutrophil recruitment and activity in the lung, thereby leading to impaired antibacterial defence and increased susceptibility to secondary infection.

To further see whether exogenous IL-17A would be sufficient to rescue influenza-infected WT mice challenged secondarily with *S. pneumoniae*, WT mice after influenza infection were treated with recombinant IL-17A protein and then challenged with *S. pneumoniae*. We found that treatment with IL-17A could significantly increase neutrophil numbers (supplementary Fig 9A) and up-regulate MPO activity in the lungs of infected mice (supplementary Fig 9B). Also, these mice treated with IL-17A had significantly reduced pneumococcal burdens (supplementary Fig 9C) and improved survival times (supplementary Fig 9D) compared to those treated with PBS control. These data were similar to those of IL-27-deficient mice treated with PBS control, thus highlighting that IL-27-mediated IL-17A inhibition in γδ T cells contributed to the susceptibility to secondary pneumococcal pneumonia.

### IL-27 mediates the inhibitory effects of IFNAR signalling on IL-17A production by γδ T cells during secondary pneumococcal infection

**Figure 5:**
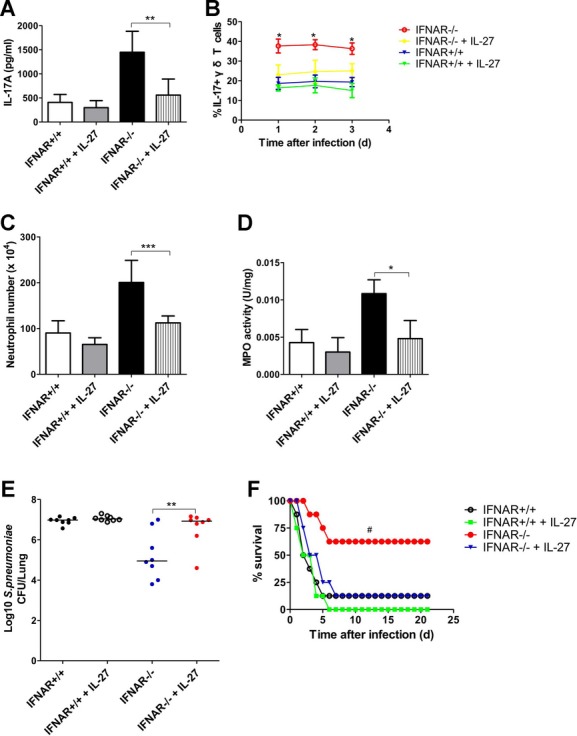
IL-27 mediated the inhibitory effects of IFNAR signalling on IL-17A production by γ´ T cells during secondary pneumococcal infection. Recombinant murine IL-27 was given i.t. into IFNAR-deficient or WT mice 24 h before intranasal pneumococcal administration, lungs were then collected for analysis at different time points. A  Lung IL-17A levels were determined at 24 h following secondary pneumococcal infection (*n* = 4). B  Percentages of IL-17A producers among γδ T cells in the lungs from groups of mice at indicated times following secondary pneumococcal challenge (*n* = 4). C  Lung neutrophil numbers at 24 h after secondary pneumococcal infection (*n* = 4). D  Lung MPO activity at 24 h after secondary pneumococcal infection (*n* = 4). E  Pulmonary pneumococcal burdens at 48 h after secondary pneumococcal infection (*n* = 8). F  Survival for IFNAR-deficient or WT mice treated with or without exogenous IL-27 after secondary pneumococcal infection (*n* = 8). *p < 0.05, **p < 0.01, ***p < 0.001 when compared between IFNAR-deficient mice treated with and without IL-27; ^#^*p* < 0.05 when compared with mice treated with IL-27.

When we prepared this manuscript, Li *et al* demonstrated that influenza-infected IFNAR-deficient mice were resistant to secondary pneumococcal pneumonia with increased IL-17A production in γδ T cells (Li *et al*, 2012), but the underlying mechanisms remained undefined. Given that IL-27 production was significantly decreased in IFNAR-deficient mice after influenza infection (Fig 3), we predicted that decreased IL-27 may be the significant mediator of enhanced clearance of secondary pneumococcal pneumonia observed in IFNAR-deficient mice, and determined whether IL-27 treatment could reverse the tolerance to secondary pneumococcal pneumonia in IFNAR-deficient mice. We found that inoculation of exogenous IL-27 could significantly down-regulate IL-17A production (Fig 5A) and the percentage of IL-17A-producing γδ T cells (Fig 5B) in the lungs of IFNAR-deficient mice after secondary pneumococcal infection. Accordingly, IL-27 treatment also significantly decreased neutrophil recruitment (Fig 5C) and MPO activity (Fig 5D) in IFNAR-deficient mice compared with control mice. In addition, IFNAR-deficient mice treated with IL-27 showed significantly higher pulmonary pneumococcal burdens (Fig 5E) and lower survival rates (Fig 5F). The pneumococcal counts and survival rates in IL-27-treated IFNAR-deficient mice were strikingly close to those of virus/*S. pneumoniae*-infected WT mice, indicating that the inhibitory effects of type I IFNs on IL-17A production by γδ T cells to promote postinfluenza pneumococcal pneumonia was mediated via IL-27. However, treatment of IL-27 did not change pneumococcal counts and survival rates in WT mice, suggesting that IL-27 was expressed at a functionally adequate level in WT mice during secondary pneumococcal pneumonia.

### IL-27 inhibits IL-17A production in γδ T cells activated by *S. pneumoniae* and DC *in vitro*

**Figure 6:**
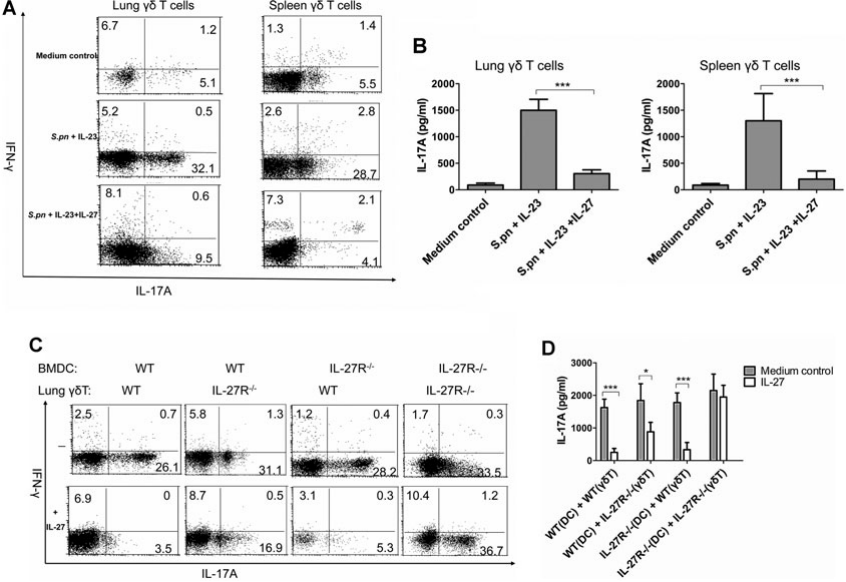
IL-27 inhibited IL-17A production by γ´ T cells in *vitro*. A  FACS analysis of IL-17A and IFN-γ expression in FACS-sorted lung or spleen γδ T cells cultured for 72 h under the stimulation of HkSp (1 × 10^8^ CFU/ml) and IL-23 (50 ng/ml) in the presence or absence of IL-27 (100 ng/ml). B  IL-17A concentrations in the supernatants of lung or spleen γδ T cells activated by HkSp and IL-23 in the presence or absence of IL-27 as determined by ELISA. C  FACS analysis of IL-17A and IFN-γ expression in FACS-sorted lung γδ T cells from IL-27R-deficient or WT mice, which were co-cultured with BMDC from IL-27R-deficient or WT mice activated by HkSp (1 × 10^8^ CFU/ml) in the presence or absence of IL-27 (100 ng/ml) for 72 h. Lung γδ T cells were gated for FACS analysis. D  IL-17A concentrations in the supernatants of lung γδ T cells from IL-27R-deficient or WT mice, which were co-cultured with BMDC from IL-27R-deficient or WT mice activated by HkSp in the presence or absence of IL-27 for 72 h. Results were from three independent experiments, and each was performed with cells isolated from three mice. *p < 0.05, ***p < 0.001 when compared between groups denoted by horizontal lines.

Functional IL-27 receptor comprises a heterodimer consisting of WSX-1 and gp130 (Hunter & Kastelein 2012; Villarino *et al*, 2003). To examine the direct effects of IL-27 on IL-17A production by γδ T cells *in vitro*, the surface expression of IL-27 heterodimer receptor on FACS-purified spleen γδ T cells or BMDC was firstly characterized by flow cytometry. As shown in supplementary Fig 10, both BMDC and γδ T cells expressed WSX-1 and gp-130, indicating that both cell populations may be responsive to IL-27. Efficient *in vitro* production of IL-17A by γδ T cells requires the combination of TLR ligands and DC-derived IL-23 (Sutton *et al*, 2009). Here we found that the combination of IL-23 and HkSp resulted in high expansion of IL-17A-secreting lung or spleen γδ T cells, and exogenous addition of IL-27 could significantly suppress IL-17A production in lung or spleen γδ T cells driven by IL-23 and HkSp (Fig 6A). ELISA also confirmed that IL-27 could significantly decrease IL-17A secretion in the supernatants of cultured lung or spleen γδ T cells (Fig 6B).

Since IL-27 receptor was expressed on γδ T cells as well as DC, it was important to determine the target cell population mediating the suppressive effects of IL-27. We therefore performed experiments using BMDC, lung γδ T cells or both from IL-27R-deficient mice. We found that IL-27 was dependent on IL-27R expression on lung γδ T cells to exert its suppressive effects, whereas its presence on DC was partially required, because the expansion of IL-17A-secreting lung γδ T cells was partially but not completely suppressed when co-culturing IL-27R-deficient lung γδ T with WT DC, while IL-27 had no inhibitory effects on the expansion of IL-17A-secreting IL-27R-deficient lung γδ T cells when co-culturing with IL-27R-deficient DC (Fig 6C). Further ELISA assays also showed that IL-17A protein secretion was partially decreased in co-culture of IL-27R-deficient lung γδ T cells with WT DC but strongly inhibited in co-culture of WT lung γδ T cells and IL-27R-deficient DC by IL-27 (Fig 6D). We further repeated our experiments using BMDC, spleen γδ T cells or both from IL-27R-deficient mice, and also found that IL-27 was dependent on IL-27R on spleen γδ T cells to inhibit IL-17A production, while its expression on DC was partially required (supplementary Fig 11A and B). Therefore, although there was somewhat variation between intracellular IL-17A in gated γδ T cells by flow cytometry and secreted IL-17A in culture supernatants by ELISA, our data clearly suggest that the suppressed production of IL-17A in γδ T cells induced by IL-27 was regulated by IL-27R on γδ T cells dominantly and at least in part via the expression of IL-27R on DC.

### IL-27 is responsible for the suppressive effects of IFN-β on IL-17A production by γδ T cells *in vitro*

**Figure 7:**
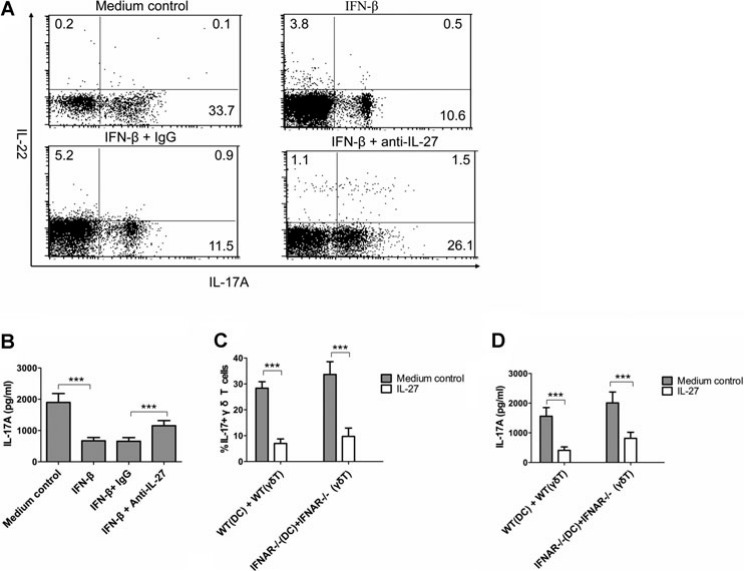
IL-27 directly contributed to IFN-b-mediated inhibition of IL-17A production in γ´ T cells. A, B  Spleen γδ T cells isolated from WT mice were restimulated with HkSp (1 × 10^8^ CFU/ml) and IL-23 (50 ng/ml) for 72 h in the presence or absence of supernatants from IFN-β-treated splenocytes plus anti-IL-27 neutralizing antibodies or control IgG. IL-17 levels were determined by (A) FACS analysis and (B) ELISA, respectively. C, D  WT or IFNAR-deficient spleen γδ T cells were co-cultured with WT or IFNAR-deficient BMDC activated by HkSp (1 × 10^8^ CFU/ml) in the presence or absence of IL-27 (100 ng/ml) for 72 h. (C) The percentage of IL-17A-producing γδ T cells was measured by FACS analysis, (D) while IL-17A secretion in the culture supernatants was detected by ELISA. Results were from three independent experiments, and each was performed with cells isolated from three mice. ***p < 0.001 when compared between groups denoted by horizontal lines.

Having shown that influenza virus induced IL-27 production in an IFNAR-dependent manner (Fig 3), and the inhibitory effects of type I IFNs on IL-17A production by γδ T cells to promote postinfluenza pneumococcal pneumonia were mediated via IL-27 *in vivo* (Fig 5), we thus determined whether IL-27 was responsible for the inhibitory effects of IFN-β *in vitro*. Our results showed that supernatants from IFN-β-treated splenocytes inhibited the development of IL-17A-producing γδ T cells stimulated by HkSp and IL-23. When anti-IL-27 antibodies were added to block the IL-27 activity in the supernatants from IFN-β-treated splenocytes, the IFN-β-mediated inhibitory effects on IL-17A production were reversed, as demonstrated by intracellular IL-17A staining (Fig 7A) and IL-17A secretion analysis (Fig 7B). On the other hand, IL-27 suppressed the development of IL-17A-producing γδ T cells from IFNAR-deficient mice (Fig 7C and D), indicating that IL-27 activity on γδ T cells was not dependent on IFNAR signalling.

### IL-27 down-regulates RORγt, IL-23R and CCR6 expression in γδ T cells and cytokine production in DC

We next examined the effects of IL-27 on the expression of RORγt, IL-23R and CCR6 in γδ T cells, which are characteristic features of IL-17-producing γδ T cells (Martin *et al*, 2009). We found that γδ T cells expressed RORγt, IL-23R and CCR6 upon co-culture with BMDC stimulated by HkSp, whereas the addition of IL-27 could significantly down-regulate the protein expression of RORγt, IL-23R and CCR6 in γδ T cells when co-cultured with BMDC stimulated by HkSp (supplementary Fig 12A).

Since previous studies have reported that IL-23 and IL-1β produced by DC played a crucial role in the induction of IL-17 from γδ T cells (Sutton *et al*, 2009), we proposed that the IL-27-induced changes in cytokine expression from DC might collectively inhibit IL-17A production by γδ T cells. As shown in supplementary Fig 12B, HkSp stimulation potently increased the production of IL-23 and IL-1β from BMDC, while IL-27 strongly suppressed the secretion of IL-23 and IL-1β by more than 80% in BMDC stimulated with HkSp, and it also significantly inhibited IL-6 production in BMDC by about 30%. These data suggest that IL-27 could down-regulate the expression of IL-23 and IL-1β in BMDC activated by*S. pneumoniae*, which was involved in decreased IL-17A production in activated γδ T cells.

### IL-27-mediated IL-17A suppression in γδ T cells is regulated by STAT1

**Figure 8:**
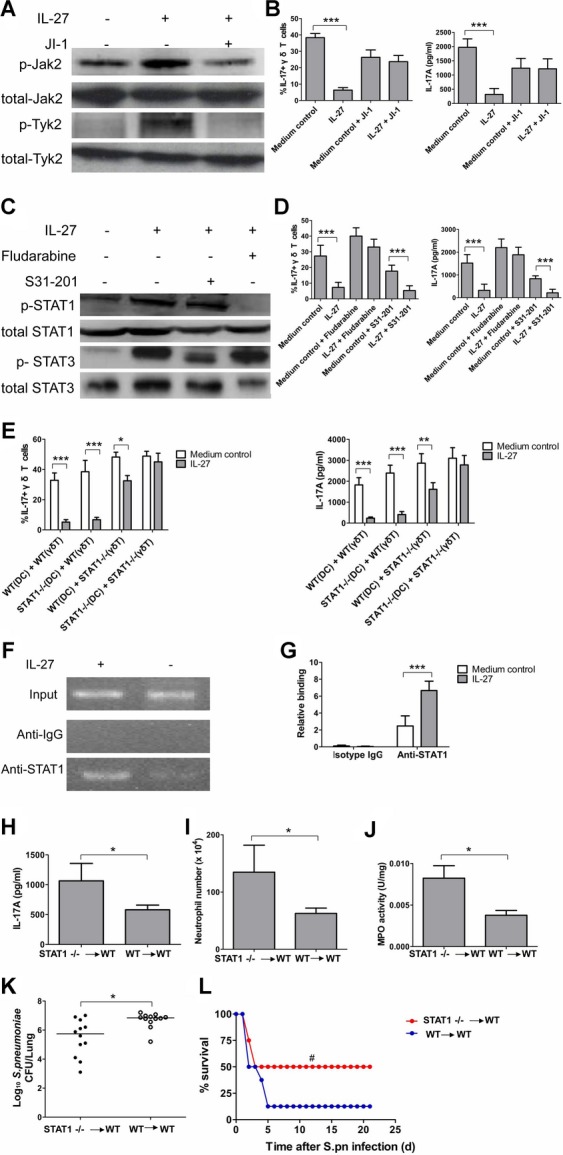
STAT1 was critical for suppression of IL-17A in γ´ T cells by IL-27 in *vitro* and in *vivo*. A  Splenocytes were pretreated with JI-1 (1 nM) for 1 h, followed by stimulation with IL-27 (100 ng/ml) for a further 15 min. Phospho-Jak2 and phosphor-Tyk2 were detected by Western blot. B  FACS analysis of percentages of IL-17A producers among γδ T cells in FACS-sorted spleen γδ T cells co-cultured with BMDC infected with HkSp (1 × 10^8^ CFU/ml) in the presence or absence of IL-27 (100 ng/ml) and of JI-1 (1 nM) for 72 h, and IL-17A concentrations in the culture supernatants were also determined by ELISA. C  Splenocytes were pretreated with fludarabine (50 μM) or S31-201 (10 μM) for 1 h, followed by stimulation with IL-27 (100 ng/ml) for a further 15 min. Phospho-STAT1 (Tyr 701) and phosphor-STAT3 (Tyr 705) were detected by Western blot. D  FACS analysis of percentages of IL-17A producers among γδ T cells in FACS-sorted spleen γδ T cells co-cultured with BMDC infected with HkSp (1 × 10^8^ CFU/ml) in the presence or absence of IL-27 (100 ng/ml) and of fludarabine (50 μM) or S31-201 (10 μM) for 72 h, and IL-17A concentrations in the culture supernatants were also determined by ELISA. E  FACS analysis of IL-17A expression in FACS-sorted spleen γδ T cells from STAT1-deficient or WT mice, which were co-cultured with BMDC from STAT1-deficient or WT mice infected by HkSp in the presence or absence of IL-27 for 72 h. The percentages of IL-17A-producing γδ T cells were determined by FACS analysis, while IL-17A concentrations were determined by ELISA. F  FACS-sorted spleen γδ T cells were stimulated with HkSp (1 × 10^8^ CFU/ml) and IL-23 (50 ng/ml) in the presence or absence of IL-27 (100 ng/ml) for 72 h. Cell lysates were immunoprecipitated either with anti-STAT1 or isotype IgG. Bound DNA was analyzed by PCR with IL-17A promoter site-specific primers. G  Eluted DNA was quantitated by quantitative PCR with primers specific for the IL-17A promoter. H  Lung IL-17A concentrations in WT mice transferred with STAT1-deficient or WT γδ T cells at 24 h after secondary pneumococcal infection (*n* = 5). I  Lung neutrophil numbers in WT mice transferred with STAT1-deficient or WT γδ T cells at 24 h after secondary pneumococcal infection (*n* = 5). J  Lung MPO activity at 24 h after secondary pneumococcal infection (*n* = 5). K  Pulmonary pneumococcal burdens at 48 h after secondary pneumococcal infection (*n* = 12). L  Survival for WT mice transferred with STAT1-deficient or WT γδ T cells after secondary pneumococcal infection (*n* = 12). *p < 0.05, **p < 0.01, ***p < 0.001 when compared between groups denoted by horizontal lines; ^#^*p* < 0.05 when compared with mice transferred with WT γδ T cells.

IL-27 mediates its biological effects mainly through JAK/STAT signalling (Hunter & Kastelein 2012). To determine the signalling pathway by which IL-27-mediated IL-17A suppression in γδ T cells, we examined Jak/STAT signalling using Jak inhibitor 1 (JI-1) (Young *et al*, 2012). At the concentration of JI-1 (1 nM) that selectively inhibited Jak2 and Tyk2 (Fig 8A), IL-27 failed to inhibit IL-17A production by γδ T cells in co-culture with BMDC stimulated by HkSp (Fig 8B), demonstrating that IL-17A suppression in γδ T cells by IL-27 was mediated by Jak2/Tyk2 activity. Since STAT1/STAT3 can be directly phosphorylated by Jak2/Tyk2 and mediates several biological functions of IL-27, we then used specific inhibitors for STAT1 (fludarabine) and STAT3 (S31-201) (Mir *et al*, 2012). At the concentration of fludarabine (50 μM) and S31-201 (10 μM) that selectively inhibited STAT1 and STAT3, respectively (Fig 8C), we found that STAT1 inhibitor fludarabine could reverse the inhibitory effects of IL-27 on the development of IL-17A-producing γδ T cells (Fig 8D). In the presence of STAT3 inhibitor S31-201, IL-27 could efficiently suppress IL-17A production by γδ T cells, which was consistent with a recent report that STAT3 was dispensable for the development of IL-17-producing γδ T cells (Shibata *et al*, 2011).

We further examined whether the suppression of IL-17A in γδ T cells by IL-27 was dependent on STAT1 using STAT1-deficient cells. In co-culture of STAT1-deficient γδ T cells and STAT1-deficient DC activated by HkSp, the suppression of IL-17A-producing γδ T cells was completely lost and IL-17A secretion was similar in the presence or absence of IL-27 (Fig 8E). In co-culture of WT γδ T cells and STAT1-deficient DC activated by HkSp, IL-27 could suppress the expansion of IL-17A-producing γδ T cells by more than 90%, while it could inhibit the expansion of IL-17A-producing γδ T cells by about 30% in co-culture of STAT1-deficient γδ T cells and WT DC. These data suggest that a dominant function for STAT1 in γδ T cells in the ability of IL-27 to antagonize IL-17A induction by γδ T cells co-cultured with DC activated by HkSp.

Initial study of the IL-17A promoter sequences demonstrated several potential STAT-binding sites within −2K bp of the IL-17A promoter (Laurence *et al*, 2007). Identification of conserved STAT1-binding site was done using the rVista 2.0 web utility. The potential STAT binding sites within −2K bp of the IL-17A promoter were located at site 1 (2 kb upstream of the transcriptional start site), site 2 (within the first intron) and site 3 (1 kb upstream of the transcriptional start site). We therefore considered the possibility that STAT1 might attenuate IL-17A production by direct binding to the IL-17A promoter in γδ T cells. To test this hypothesis, we stimulated γδ T cells with BMDC activated by HkSp in the presence or absence of IL-27. As shown in Fig 8F, strong specific STAT1 binding was detected at IL-17A promoter in the presence of IL-27, which was further confirmed by quantitative PCR (Fig 8G).

The *in vivo* physiological relevance of the STAT1-dependent IL-17A suppression in γδ T cells by IL-27 was further investigated in a model of postinfluenza pneumococcal pneumonia. We adoptively transferred WT or STAT1-deficient γδ T cells into WT mice following primary influenza infection, and then mice were challenged with secondary pneumococcal infection. We found that STAT1 deficiency in γδ T cells enhanced IL-17A production (Fig 8H), and increased neutrophil recruitment (Fig 8I) as well as up-regulated MPO activity (Fig 8J) in the lungs of mice. Furthermore, STAT1 deficiency in γδ T cells improved the ability of mice to clear pneumococcus (Fig 8K), and mice receiving STAT1-deficient γδ T cells had a significantly lower mortality when compared with that of mice receiving WT γδ T cells (Fig 8L). Therefore, the ability of IL-27-mediated IL-17A suppression in γδ T cells to sensitize mice to secondary pneumococcal pneumonia was dependent upon STAT1.

### IL-27 inhibits IL-17A production in human γδ T cells activated by *S. pneumoniae* and DC

**Figure 9:**
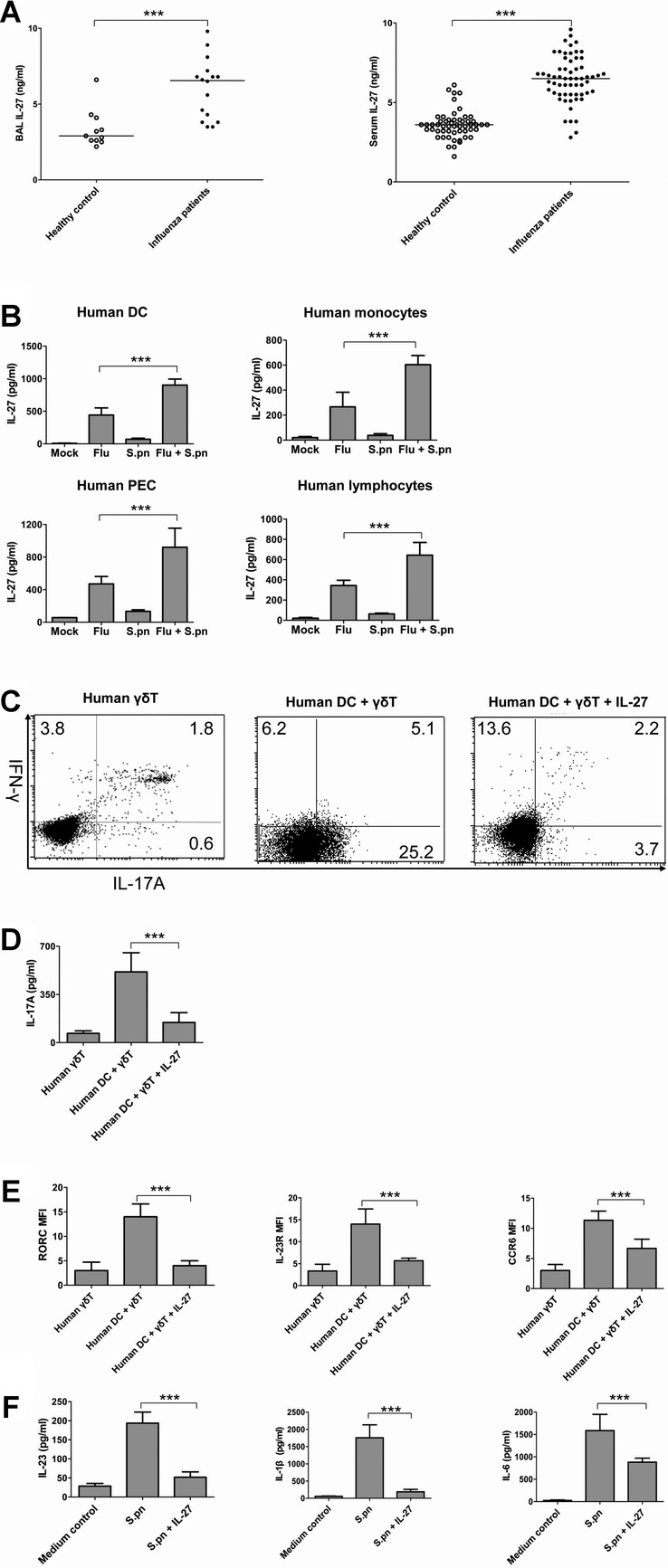
IL-27 inhibited IL-17A production in human Vγ9V´2 T cells. A  ELISA analysis for IL-27 levels in BAL and serum samples from healthy individuals and influenza-infected patients. B  ELISA analysis for IL-27 production in human cells. Human monocyte-derived DC, momocytes, pulmonary epithelial cells and lymphocytes were stimulated with influenza virus (MOI = 1) and HkSp (1 × 10^8^ CFU/ml). After 24 h, ELISA was performed to measure the IL-27 concentrations in the culture supernatants. C  FACS analysis for IL-17A and IFN-γ expression in human Vγ9Vδ2 T cells co-cultured with DC activated by HkSp (1 × 10^8^ CFU/ml) in the presence or absence of IL-27 (100 ng/ml) at 5 days. D  IL-17A concentrations in the supernatants of human Vγ9Vδ2 T cells co-cultured with DC activated by HkSp in the presence or absence of IL-27 at 5 days. E  The expression of RORγt, IL-23R and CCR6 in human Vγ9Vδ2 T cells co-cultured with DC infected with HkSp in the presence or absence of IL-27 F  ELISA analysis for cytokine production from human DC activated by HkSp in the presence or absence of IL-27. Results were from three independent experiments, and each was performed with cells isolated from three different donors. ***p < 0.001 when compared between groups denoted by horizontal lines.

To translate findings from murine to human systems, we investigated whether IL-27 could suppress IL-17A production in human γδ T cells. Firstly, we found that IL-27 levels in the bronchoalveolar lavage (BAL) and serum samples from influenza-infected patients were significantly elevated compared with control individuals (Fig 9A). Influenza infection could also potently induce IL-27 production in human monocyte-derived DC, monocytes, pulmonary epithelial cells and lymphocytes, while HkSp further enhanced the effects of influenza virus on IL-27 secretion (Fig 9B).

To test the effect of IL-27 on IL-17A production by human Vγ9Vδ2 cells, highly purified human Vγ9Vδ2 T cells were co-cultured with autologous DC activated by HkSp and isopentenyl pyrophosphate (IPP), in the presence or absence of IL-27, and then incubated for 5 days in the presence of low doses of IL-2 and re-stimulated for 6 h with IPP and brefeldin A. Intracellular cytokine staining assay showed that IL-27 down-regulated the increased percentage of IL-17A-producing human Vγ9Vδ2 cells (Fig 9C), and similar results were obtained by measuring IL-17A secretion in culture supernatants (Fig 9D). IL-27-mediated inhibition of IL-17A production in Vγ9Vδ2 T cells was also associated with significantly reduced expression of the human orthologs of mouse RORγt (RORC), IL-23R and CCR6 (Fig 9E). Furthermore, IL-27 could significantly decrease the up-regulated production of IL-23, IL-1β and IL-6 from human DC stimulated by HkSp (Fig 9F). Collectively, these data indicate that IL-27 has the ability to inhibit the production of IL-17A in antigen-primed human Vγ9Vδ2 T cells.

## Discussion

Neutrophil response is an essential component of host innate immunity against pneumococcal lung infection (Lu *et al*, 2008; Paton & Ferrante^1983^). In this study, we found that γδ T cells, but not αβ T cells, were required for neutrophil recruitment and activity in host defence against pneumococcal infection via IL-17A production. Importantly, we reported a novel mechanism by which IL-27, an immunosuppressive cytokine downstream of IFNAR signalling pathway, impaired innate immune response against secondary pneumococcal challenge by suppressing IL-17A production in γδ T cells in a STAT1-dependent manner, which in turn inhibited neutrophil response. These findings indicate that IL-27 signalling activated after influenza infection could promote the development of secondary pneumococcal pneumonia.

IL-17A plays an important role in orchestrating innate immunity by regulating granulopoiesis and neutrophil accumulation in peripheral tissues for pathogen clearance (Rendon & Choudhry 2012). CD4+ T cells have been considered to be the major source of IL-17A. Nevertheless, this adaptive Th17 response develops slowly. Recently, it has been found that there are innate IL-17A producers that contribute to the first line of defence against pathogens that require IL-17A-mediated neutrophil response (Sutton *et al*, 2012; Zeng *et al*, 2012). Among these, γδ T cells have the inherent ability to very rapidly produce IL-17A as an essential component of the innate immune response, and γδ T-cell-derived IL-17A is one of the earliest sources of this cytokine after infection in mucosal tissues (Powolny-Budnicka *et al*, 2011). In fact, γδ T cells are committed to IL-17A production already in the thymus (Haas *et al*, 2012), and direct pathogen recognition by γδ T cells via TLRs leads to initial IL-17A production, which is stabilized and expanded by IL-23 secreted by antigen-presenting cells (APC) in response to pathogen recognition (Martin *et al*, 2009). Besides, γδ T-cell-derived innate IL-17A has been demonstrated to promote adaptive IL-17A production in Th17 cells, suggesting an addition role of γδ T-cell in host defence and inflammatory diseases (Sutton *et al*, 2009). Our current study demonstrated a dominant role for γδ T cells in stimulating neutrophil response via innate IL-17A production against pneumococcal lung infection. However, influenza virus infection selectively attenuated innate IL-17A production by γδ T cells upon secondary pneumococcal infection, which contributed to the development of postinfluenza pneumococcal pneumonia.

Since influenza infection was associated with profoundly elevated IL-27 production in the lungs of mice, we investigated whether the induction of IL-27 was responsible for altering innate immunity in such a way that secondary pneumococcal replication was enhanced. In the mouse model of postinfluenza pneumococcal pneumonia, IL-17A production in γδ T cells was significantly increased in IL-27R-dificient mice relative to WT mice. The increased IL-17A production by γδ T cells was associated with up-regulated neutrophil response, and decreased lung pneumococcal burdens as well as improved survival in IL-27R-dificient mice. Furthermore, treatment with anti-IL-27 blocking antibodies could protect against secondary pneumococcal infection, indicating that immunomodulatory therapy aimed at antagonizing IL-27 may provide a potential therapeutic intervention for humans susceptible to postinfluenza pneumococcal pneumonia. Although pneumococcal infection alone was associated with some increase in IL-27 production, yet this response did not seem to be sufficient to impact pneumococcal infection, since lung pneumococcal burdens were unaltered in IL-27R-deficient mice.

Recently, it has been demonstrated that influenza-infected IFNAR-deficient mice were resistant to secondary pneumococcal pneumonia with increased IL-17A production in γδ T cells (Li *et al*, 2012). Nevertheless, the means by which type I IFNs could modulate innate immunity during postinfluenza pneumococcal pneumonia remain unknown. Here we showed that influenza induced IL-27 production in an IFNAR-dependent manner, and exogenous administration of IL-27 could reverse the resistance phenotype in IFNAR-deficient mice upon postinfluenza pneumococcal infection via down-regulating IL-17A production in γδ T cells and neutrophil response. These results suggest that the inhibitory effects of IFNAR signalling on IL-17A production by γδ T cells during secondary pneumococcal infection rely on IL-27. Therefore, we reported what we believe to be a novel negative role of IL-27 in IL-17A production by γδ T cells upon secondary pneumococcal infection. This might provide a molecular basis for the interesting findings reported by Wirtz *et al*, who described that mice deficient for the EBI3 subunit of IL-27 displayed significantly enhanced neutrophil migration, which resulted in enhanced *Escherichia coli* clearance and local control of infection in septic peritonitis (Wirtz *et al*, 2006), because IL-17 production in γδ T cells was also important for local neutrophil influx and protection against*E. coli* (Nakamura *et al*, 2008).

We also analyzed the mechanism of IL-27-mediated IL-17A suppression in γδ T cells *in vitro*. This negative effect of IL-27 on IL-17A production was found to be strictly dependent on IL-27R on γδ T cells, whereas its presence on DC played a partial role. It has been demonstrated that IL-23 and IL-1β from DC were implicated in promoting IL-17A production in γδ T cells (Sutton *et al*, 2009), while IL-6 was dispensable (Lochner *et al*, 2008). Our resulted showed that IL-27 strongly suppressed the secretion of IL-23 and IL-1β in DC activated by HkSp. These observations suggest that in addition to a direct inhibitory effect of IL-27 on IL-17A production in γδ T cells, IL-27 could suppress IL-23 and IL-1β secretion from DC activated by *S. pneumoniae*, thereby leading to IL-17A inhibition in γδ T cells indirectly.

Evidence in support of that IL-27 mediated the inhibitory effects of IFNAR signalling on IL-17A production in γδ T cells during secondary pneumococcal infection was further provided by our demonstration that neutralization of IL-27 abrogated the inhibitory effects of IFN-β on the development of IL-17A-pruducing γδ T cells *in vitro*. Since early expression of type I IFNs is a molecular signature of influenza virus infection (Katze *et al*, 2002; Shinohara *et al*, 2008), which regulates IL-27 production upon influenza infection as observed in this study, we can conclude that IL-27 signalling occurs downstream of type I IFNs in the suppression of IL-17A production by γδ T cells, thereby promoting secondary pneumococcal infection.

IL-27 has been reported to activate JAK/STAT signalling in αβ T cells (Kamiya *et al*, 2004). We have recently shown the involvement of p38MAPK, PI3K-Akt and NF-κB in regulating cytokine expression in lung epithelial cells and fibroblasts activated by IL-27 (Cao *et al*, 2012; Dong *et al*, 2013). Here we demonstrated a dominant role of STAT1 in regulating IL-27-mediated suppression of IL-17A production in γδ T cells, and enhanced binding of STAT1 to the IL-17A promoter was observed after IL-27 stimulation in γδ T cells, indicating that STAT1 is a negative signal for IL-17A expression in γδ T cells. Furthermore, adoptive transfer of STAT1-deficient γδ T cells to influenza-infected WT mice could restore the role of γδ T cells in combating secondary pneumococcal infection by up-regulating IL-17A production, suggesting an additional way for treating postinfluenza pneumococcal pneumonia by antagonizing STAT1.

Although multiple studies have addressed the basis for the inhibitory effects of IL-27 on Th1, Th2 and Th17 cell responses (Banchereau *et al*, 2012; Hunter & Kastelein 2012), this is the first study linking IL-27 to IL-17A suppression in innate γδ T cells, which sensitized the host to secondary pneumococcal pneumonia following influenza infection. Mechanistically, it has been proposed that IL-2 may serve to limit immune responses in certain circumstances (Laurence *et al*, 2007). We did not find evidence of altered IL-2 production in IL-27R-deficient mice with postinfluenza pneumococcal pneumonia when compared with WT mice (data not shown). Another immunosuppressive action of IL-27 is mediated by IL-10 production, however, several independent research groups have demonstrated that there was no significant difference between WT and IL-10-deficient mice in susceptibility to secondary pneumococcal infection (Shahangian *et al*, 2009; Sun & Metzger 2008), indicating that IL-27-mediated IL-10 expression is unlikely to be responsible for the regulatory effects of IL-27 on antibacterial immunity in our model. Besides, although IL-17A production by Th17 cells was limited in the current study, this does not preclude the possibility that IL-27 may also contribute to aggravating the pathogenesis of postinfluenza pneumococcal pneumonia by inhibiting Th17 development at later time-points. Ongoing studies should continue to identify additional suppressive functions of IL-27 over time depending on the progress of secondary pneumococcal pneumonia after influenza infection.

In summary, influenza infection induced IL-27 production in an IFNAR-dependent pathway, which suppressed innate IL-17A production by γδ T cells upon secondary pneumococcal infection in a STAT1-dependent manner, thereby promoting the development of secondary pneumococcal pneumonia. The fact that IL-27 neutralization or IL-17A administration could restore anti-pneumococcal effects opens a new door for protecting human population from secondary pneumococcal infections especially during influenza pandemics.

## Materials and Methods

### Mice

C57BL/6 mice aged 6–8 weeks were obtained from and raised at Chongqing Medical University. IL-27R^−/−^ (WSX-1-deficient), IFNAR^−/−^ and γδ TCR^−/−^ mice raised on C57BL/6 background were purchased from The Jackson Laboratory, while STAT1^−/−^ mice were from Taconic Transgenics. All mice were housed under humidity- and temperature-controlled specific pathogen-free conditions in the animal facility of Chongqing Medical University. All animal experiments were done in accordance with the Institutional Animal Care and Use Committee's guidelines at the Chongqing Medical University.

### Infectious reagents and mice infection

Influenza virus strain A/PR/8/34 (H1N1, ATCC) was grown on MDCK cells. Virus was harvested by a freeze/thaw cycle, followed by centrifugation at 680 × *g* for 10 min. Supernatants were stored in aliquots at −80°C. Titration was performed to calculate the median tissue culture infective dose (TCID50) of the viral stock. Type 3 *S. pneumoniae* (ATCC 6303 clinical isolate with capsular serotype 3) were grown in Todd-Hewitt broth with yeast extract at 37°C for 8 h or until log phase. The concentration of bacteria in broth was determined by measuring the absorbance at 600 nm.

Mice were infected using an Inhalation Exposure System (Glass-Col, USA) for influenza virus infection, a dose of 200 PFU of influenza A PR/8/34 H1N1 (in 40 μl sterile PBS) from a frozen stock or control PBS was given. Body weight and viral PFUs in lung homogenates of influenza-infected mice were then assessed. For *S. pneumoniae* infection, mice were anaesthetized with pentobarbital sodium intraperitoneally (i.p.) (30 mg/kg weight), and then 5000 CFU *S. pneumoniae* in 30 μl sterile PBS was administered intranasally into mice as described in our previous studies (Gong *et al*, 2011), which mimicked the natural route of pneumococcal infection.

### Patient samples

BAL and serum samples were collected from 55 healthy individuals and 61 patients who were confirmed influenza infection in 2009 H1N1 pandemic according to a standardized protocol, as recommended by task force guidelines of the American Thoracic Society, as described previously (Cao *et al*, 2012; Dong *et al*, 2013). This protocol was approved by the Clinical Research Ethics Committee of The First Affiliated Hospital of Chongqing Medical University, and informed consent was obtained from all participants according to the Declaration of Helsinki.

### Reagents

Recombinant murine or human IL-4, IL-17A, IFN-β, GM-CSF, IL-23, IL-27, anti-mouse IL-17A and anti-mouse IL-27p28 antibodies were purchased from R&D Systems (Minneapolis, MN). Heat-killed *S. pneumoniae* was obtained by boiling 1 × 10^8^ CFU in phosphate-buffered saline (PBS) for 20 min and checking for viability by colony counts and plate streaking. Fludarabine was purchased from Sigma–Aldrich (St. Louis, MO), while S31-201 and JI-1 were from Calbiochem (Merck KGaA, Germany).

### Cell culture

Preparation of murine monocytes and BMDC was conducted according to methods described in the previous study (Lutz *et al*, 1999). Murine lung epithelial cells (LEC) were isolated as described previously (Kim *et al*, 2011; You *et al*, 2002). Briefly, lungs were perfused with 20 ml of sterile PBS via the right ventricle until they were visually free of blood, and then filled (2 ml per lung) via the airway with RPMI 1640 with 2.5% FBS (HyClone Laboratories), 80 U elastase and 0.05 mg/ml trypsin (Sigma–Aldrich). After incubation at 37°C for 25 min, the lungs were homogenized. Then the homogenate was centrifuged at 2000 rpm for 2 min, and the supernatant fraction containing the cell suspension was layered on top of an isotonic Percoll solution (1.082 g/ml, GE Healthcare, Little Chalfont, U.K.) and centrifuged for 25 min at 1500 rpm at 4°C. The cells at the interface between Percoll layers were removed and cultured in plates coated with anti-Fc receptor mAbs (BD Pharmingen) at 37°C for 30 min. Then the nonadherent cells were collected, centrifuged and resuspended in DMEM containing with 10% FBS and 1% penicillin–streptomycin. Finally, these resuspended cells were cultured to >90% confluence in DMEM containing with 10% FBS for future experiments. The purity of these isolated LEC was identified using anti-mouse pan-cytokeratin mAbs (Abcam) and anti-mouse FcgIII/II receptor mAbs (BD Pharmingen). Flow cytometric analysis showed the purity of LEC was >90%.

For generation of human monocyte-derived DCs, PBMC were isolated from the buffy coat of healthy volunteers using Ficoll-Paque (Pharmacia Biotech, Uppsala, Sweden) density gradient centrifugation. CD14+ monocytes were purified from these cells by the MACS system (Miltenyi Biotec, Auburn, CA, USA). The generation of human DC was performed according to our previous study (Cao *et al*, 2013). Primary human pulmonary epithelial cells (PEC) were purchased from ScienCell Research Laboratories, and they were cultured in serum-free pulmonary epithelial cell medium as described in our study (Cao *et al*, 2010).

### γδ T-cell purification and cell sorting

Murine γδ T cells from spleens or lungs were positively selected by indirect labelling with anti-γδ TCR MicroBeads (Miltenyi Biotec) or MACS followed by FACS sorting with APC-conjugated anti-γδ TCR (clone GL3) mAbs from BD Biosciences on a FACSAria flow cytometer (BD Biosciences). Human peripheral Vγ9Vδ2 cells were purified by MACS using Vδ2-specific microbeads (Miltenyi Biotec) and confirmed by FACS. The final purity of isolated murine or human γδ T cells was ∼99%.

### Quantification of influenza virus or *S. pneumoniae* in the lung

At the designated time points, mice were euthanized by i.p. pentobarbital, and then whole lungs were removed and homogenized in 1 ml of PBS supplemented with protease inhibitor cocktail (Roche Applied Science). Plaque assays for viral titration were performed by incubating MDCK monolayers in 6-well plates at 37°C for 1 h with whole-lung homogenates which were serially diluted in virus dilution buffer, and then the infected monolayers were added with viral growth medium and incubated for 72 h at 37°C 5% CO_2_. The numbers of influenza virus plaques were counted by crystal violet staining. Pneumococcus burden was determined in the whole lung by serially diluting1:5 in PBS and plated on blood agar to determine lung CFU.

### Pulmonary histopathology and cell isolation in the lung

Formalin-fixed, paraffin-embedded 6-μm sections of lungs were used for immunohistochemistry by Haematoxylin and Eosin (H&E) staining, and cytospins were prepared for determination of differential cell counts in the lungs using a modified Wright stain. For isolation of different cells, single cell suspensions in the homogenized lungs were treated with red blood cell lysis buffer (BD Biosciences). Anti-CD4 (L3T4), anti-CD8 (Ly-2), anti-CD11b (M1/70), anti-γδ TCR (GL3), anti-NK1.1 (PK136) mAbs were then used to bind CD4+, CD8+, CD11b+ cells, γδ T and NKT cells, and cell populations were purified following manufacturer's instruction of anti-biotin magnetic microbeads (Miltenyi Biotech) and sorted on a FACSAria flow cytometer (BD Biosciences).

### MPO assay

MPO enzymatic activity was determined from homogenized lungs using an MPO assay kit according to the manufacturer's recommendations (Cytostore). The amount of MPO activity was normalized to the weight of the lung specimen, and data were presented as U/mg tissue.

### Quantitative real-time RT-PCR

Total cellular RNA was extracted using TRIzol (Invitrogen) and reversed transcribed into cDNA (Roche, USA). qPCR reactions based on SYBR green detection were performed using ABI PRISM 7500 sequence detection system (Applied Biosystems). All reactions were normalized to GAPDH. Primers for target genes were described in supplementary Table 1. Quantitative comparison was obtained through the ΔΔCT method.

### ELISA

IL-1β, IL-6, IL-17, IL-21, IL-22, IL-23, IL-27, CXCL1, CXCL10, G-CSF and TNF-α ELISA kits were purchased from R&D Systems. For lung homogenates, whole lungs were removed, taking care to dissect away lymph nodes. The lungs were homogenized in 1 ml of PBS supplemented with protease inhibitor cocktail (Roche Applied Science), followed by centrifugation at 1000 × *g* for 10 min. Supernatants were stored at −80°C until further use. Cytokine/chemokine/growth factor in total lung lysates was measured by ELISA according to the manufacturer's protocol. For human BAL and sera samples, cytokine/chemokine was also determined with commercially available ELISA kits from R&D Systems as described in our previous studies (Cao *et al*, 2012; Dong *et al*, 2013).

### Western blot analysis

Western blot analysis was done as described previously (Dong *et al*, 2013). Briefly, murine splenocytes (5 × 10^6^ cells) were washed with ice-cold PBS, and lysed in lysis buffer after treatment. Thirty micrograms of protein samples were subjected to 10% SDS–PAGE before blotting onto a polyvinylidene difluoride membrane (Amersham and Pharmacia Biotech). After blocking with 5% nonfat dry milk, membranes were incubated with mAbs to total or phospho-Jak2, total or phospho-Tyk2, total or phospho-STAT1-Tyr 701, total or phospho-STAT3-Tyr 705 (Cell Signaling Technology). Antibody–antigen complexes were finally detected using an enhanced chemiluminescent detection system according to the manufacturer's instructions (Amersham and Pharmacia Biotech).

### Flow cytometric analysis of cell-surface expression of cytokine receptors

DC or γδ T cells were incubated with anti-WSX1 (R&D Systems), or anti-gp130 (R&D Systems) mAbs. In another setting, γδ T cells were stained with anti-IL-23R (R&D Systems), or anti-CCR6 (BD Pharmingen), and their corresponding mouse IgG isotype (R&D Systems) at 4°C for 60 min in dark. After final washing, cells were resuspended in 1% paraformaldehyde in PBS. Expression of surface molecules on 5000 viable cells was then quantitatively analyzed by flow cytometry (FACSCalibur flow cytometer; BD Biosciences) in terms of mean fluorescence intensity (MFI).

### Flow cytometric analysis of intracellular molecules

For intracellular staining, single cell suspensions were restimulated with 500 ng/ml phorbol dibutyrate (PdBU) and 500 ng/ml ionomycin in the presence of 5 μg/ml brefeldin A (Sigma–Aldrich), and then cells were blocked with Fcγ blocker (BD Pharmingen) and stained with antibodies against extracellular markers (anti-CD3, anti-CD4, anti-CD8, anti-γδ TCR or anti-NK1.1; BD Pharmingen). Cells were then incubated with Fix/Perm solution (BD Pharmingen) for 20 min before washing in Perm/Wash buffer (BD Pharmingen). Staining with mAbs against intracellular cytokines was performed with anti-IL-17A (BD Pharmingen), anti-IFN-γ (BD Pharmingen) and anti-IL-22 (BD Pharmingen) or anti-RORγt (eBioscience) antibodies diluted in Perm/Wash buffer. After staining, cells were washed and resuspended in PBS 3% (v/v) FCS prior to FACS analysis.

### Chromatin immunoprecipitation (CHIP)

CHIP was conducted as previously described (Ichiyama *et al*, 2008). Briefly, DNA-bound transcription factors in treated-γδ T cells were crosslinked by infusing complete medium containing 1% formaldehyde for 10 min followed by sonication of cell lysates to shear DNA. After preclearing with protein A agarose beads (Upstate), cell lysates were immunoprecipitated with specific anti-STAT1 antibodies or rabbit control IgG overnight at 4°C. After washing and elution, crosslinks were reversed at 65°C for 4 h. The eluted DNA was then purified and analyzed by either relative-PCR or quantitative-PCR with IL-17A promoter site-specific primers. The binding to STAT1 sites in the IL-17A promoter region was performed using the following primers: GGA GAG ATG GCT CAG CAG TTA AG; reverse primer, TGG TTT CTG GGA ATT GAA CTC A. The *Ct*value of each sample was normalized to the corresponding input value and expressed as fold induction relative to the normal rabbit serum control, which was calculated as 1.0.

### Antibody-mediated neutralizations and cytokine reconstitutions

IL-27 neutralization was performed by i.p. administration of 1.0 mg of anti-IL-27 antibodies on day 0 (same day as influenza virus infection), followed by booster doses of 0.5 mg on days 2 and 4. Normal mouse IgG was used as a control. On day 5, mice were infected with *S. pneumoniae* intranasally. For neutralization of IL-17A, 1.5 mg of neutralizing rabbit anti-mouse IL-17A antibodies was administered i.p. into mice followed by infection with *S. pneumoniae* intranasally. Control rabbit IgG was used as an isotype control. For IL-17A reconstitutions, influenza-infected mice were treated i.t. with a single dose of IL-17A (10 μg) resuspended in 0.1% BSA in PBS followed by intranasal pneumococcal challenge closely. For IL-27 reconstitutions, influenza-infected mice were treated i.t. with a single dose of IL-27 (5 μg), and then mice were intranasally infected with *S. pneumoniae* 24 h later. The IL-17A or IL-27 contained less than 1.0 U endotoxin/1 μg cytokine, as determined by Limulus amoebocyte lysate assay (sensitivity limit 12 pg/ml; Biowhittaker, Inc., Walkersville, MD). Mock-treated animals received PBS and 0.1% BSA. Pulmonary pneumococcal burdens and lung homogenate MPO activity were assessed at indicated times.

### Adoptive transfer experiments

γδ T cells were purified from the whole splenocytes of naive C57BL/6, IL-27R^−/−^ or STAT1^−/−^ mice as described above. Then 1.5 × 10^6^ γδ T cells in 50 μl PBS or control PBS were adoptively transferred i.t. into naïve or influenza-infected mice. The mice were anaesthetized and hung by their large front teeth. The tongues were pulled aside and purified γδ T cells were instilled into the trachea by a syringe with flexible needle, and mice were then infected intranasally with *S. pneumoniae* 24 h later.

### Statistics

Differences between the overall survival rates for groups of mice were analyzed by Fisher exact test. For other data, statistical significance was determined using 2-tailed unpaired *t*-test, 2-tailed Mann–Whitney *U*-test (for CFU Data), or 1-way ANOVA with Bonferroni's multiple comparison post hoc tests where appropriate. A *p*-value of 0.05 or less was considered statistically significant. All calculations were performed using the Prism software program (GraphPad Software, Inc.). All bars across CFU data are presented as medians of the biological replicates. Error bars in all graphs indicate SD and represent biological replicates.

## Author contributions

The study was conceived by JC, YY and DW. JC, YY, GR, YX and LZ designed the study and analyzed the data. JC, DW, FX, YG, HW, ZS, DagLi, HZ, DaiLi, HX, XL, SL, YD and XZ performed the experiments. JC and YG wrote the manuscript with contributions of DW, YY, YD and GR. All authors read and approved the manuscript.
