# Supplementary material for: Activation of IL-27 signalling promotes development of postinfluenza pneumococcal pneumonia
Source: EMBO Mol Med. 2013 Oct 29;6(1):120–40. doi: 10.1002/emmm.201302890 (PMC3936494; doi:10.1002/emmm.201302890)
Supplement: Supplementary file 1 [file emmm0006-0120-sd1.pdf]

EMBO Molecular Medicine

Activation of IL-27 signaling promotes development of postinfluenza pneumococcal pneumonia

Ju Cao, Dongsheng Wang, Fang Xu, Yi Gong, Hong Wang, Zixin Song, Dageng Li, Hua Zhang, Dairong Li, Liping Zhang, Yun Xia, Huajian Xu, Xaiofei Lai, Shihui Lin, Xuemei Zhang, Guosheng Ren, Yubing Dai, Yibing Yin

Corresponding author: Ju Cao, Chongqing Medical University and Yibing Yin, Chongqing Medical University

---

Review timeline:

Submission date:	12 April 2013
Editorial Decision:	08 May 2013
Revision received:	26 August 2013
Editorial Decision:	06 September 2013
Revision received:	19 September 2013
Accepted:	19 September 2013

Transaction Report:

(Note: With the exception of the correction of typographical or spelling errors that could be a source of ambiguity, letters and reports are not edited. The original formatting of letters and referee reports may not be reflected in this compilation.)

Editor: Céline Carret

1st Editorial Decision 08 May 2013

Thank you for the submission of your manuscript to EMBO Molecular Medicine. We have now heard back from the two referees whom we asked to evaluate your manuscript. Although the referees find the study to be of interest, they also raise a number of concerns that need to be addressed in a major revision of your article.

As you will see from the comments below, both referees agree on the quality of the presented data and overall novelty and impact of the study. However, they also suggest strengthening the mechanistic insights and provide helpful suggestions to solidify the conclusions that I would kindly encourage you to follow. In addition, they also require more details and text clarifications to improve the readability of the article.

Should you be able to address these criticisms in full, we would be willing to consider a revised manuscript, with the understanding that acceptance of the manuscript would entail a second round of review.

Please note that that it is our journal's policy to allow only a single round of revision, and that acceptance or rejection of the manuscript will therefore depend on the completeness of your response and the satisfaction of the referees with it.

EMBO Molecular Medicine has a "scooping protection" policy, whereby similar findings that are published by others during review or revision are not a criterion for rejection. Should you decide to

submit a revised version, I do ask that you get in touch after three months if you have not completed it, to update us on the status.

Please also contact us as soon as possible if similar work is published elsewhere. If other work is published we may not be able to extend the revision period beyond three months.

I look forward to receiving your revised manuscript.

***** Reviewer's comments *****

Referee #1 (Comments on Novelty/Model System):

The studies appear to be appropriately done and analyzed in an appropriate mouse model, with verification of important effects in human samples.

Referee #1 (Remarks):

This manuscript by Cao et al presents a thorough and well-crafted investigation of the role of IL-27 and IL-17 on post-flu susceptibility to S. pneumo pneumonia. Their findings suggest that IL-27 induction after flu results in decreased gdT cell-derived IL-17 in response to S. pneumo that mediates decreased neutrophil-mediated protection. Neutralization experiments in mice and verification of IL-27 induction and effects in human samples provide translational relevance. My enthusiasm for this manuscript is high, but I feel it could be further strengthened as noted in my comments below:

1. The results suggest flu may act on a variety of cell types to induce IL-27,which then has JAK2/STAT1-dependent suppression of IL-17 in gamma delta T cells. A missing link here is how flu is inducing IL-27, especially in the context of the recognized immunosuppressive effects of flu-induced type I IFN. Is induction of IL-27 triggered by type I IFN? This could be addressed by using IFNAR KO mice/cells. The authors mention in the discussion unshown data that uses anti-IFNa Ab, but given the numerous species of type I IFN, this seems an inadequate approach.

2. The authors cite a recent manuscript by Li et al. that implicated gdT cell IL-17 suppression by type I IFN. The authors should more explicitly put their work in context of this. In particular, they should investigate/discuss whether any STAT-1 inducer suppresses IL-17 and whether the effects of type I IFN and IL-27 on gdT cell IL-17 are independent of each other.

3. Much important data has been put in supplemental materials. If at all possible, some of this should be put in the main figures. For example, the human data in Fig S5 and the establishment of the role of IL-17 in Fig S1. If needed due to space limitation, some of the 'negative' data, for example on non-IL17 cytokines in Fig 2 etc, as well as the 27R expression data in Fig 5, could be moved to supplemental instead. On a similar note, Fig 2F showing the CFU and mortality data in the context of flu should be moved to the beginning of this figure to establish their model--and then this can be followed by the cytokine analysis etc.

4. The flow cytometry evaluation of gamma delta T cells should be presented in more detail as supplemental info. For example, in Fig. 1, plots of gamma delta vs. CD45 or total cells should be shown to provide confidence that gating is being done appropriately. Documentation of purity of sorted cells with flow plots should be provided.

5. The CFU difference between 27R wt and ko in Fig 3D-E seems very modest (less than a log difference), yet it results in dramatic survival differences. Is this expected, or are other IL-27-mediated host factors contributing to the mortality effects? The correlation between small CFU differences and mortality should be discussed in the context of the authors' experience with this system or by citing appropriate literature. Also, statistics are not provided for survival curves, e.g. Fig 3D-E.

6. In Fig 4/S4, there is nice use of adoptive transfer experiments and recombinant cytokines to

establish the role of cell subtypes and cytokines. Use of neutralizing antibody against IL-17 in 27R KO mice would solidify the role of IL-17 in mediating the effects of IL-27.

7. In Fig 5D-E, the DC-gdT coculture system nicely addresses the role of these cell subsets. Including conditions where both subsets are 27R-deficient would clarify whether this would account for the full effect of 27R deficiency on IL-17. If not, the potential reasons should be discussed. In addition, 27R-deficiency in gdT cells almost fully accounted for the 17 suppression by flow but only partially by ELISA--how do the authors account for this? Finally, the authors should clarify if the 17 data reflects gating on gdT cells or not.

8. Fig 7: specific activity of the Jak inhibitor should be shown/referenced. If possible, the effect of STAT3 deficiency in comparison to STAT1 deficiency should be shown. Why isn't mortality shown for the adoptive transfer experiment?

9. Better explanation/rationale for each set of experiments would be helpful--for example, rationale for looking at IL-27 in Fig 3, explaining that gp130 and WSX-1 are 27 receptors in Fig 5.

10. For Fig 2C and similar graphs, I would suggest sticking with the convention and putting the independent variable (IL17) on the y axis instead of the x axis--this would also make it consistent with the rest of the graphs.

11. Better explanation of ELISA on lung homogenates in the methods is needed (how much material was tested, how was it done).

Referee #2 (Remarks):

Cao et al show here that influenza infection leads to high lung IL-27 levels which are able to block IL-17 induction in gd T cells, leading to lower neutrophil counts, higher bacterial load and higher susceptibility upon secondary bacterial infection. This process is STAT1 dependent and also active in human gd T cells. Overall, this study is well performed and controlled, with amazingly clear data. Many of the concerns as listed below refer to clarity of expression, which sometimes lacks to the point of rendering comprehension impossible.

Major concerns:

1. General concern: There is similar, published data (which the authors also quote) showing that IFNab suppresses IL17 from gd T cells - so this m/s appears to delineate a parallel mechanism. The relationship between IFNab and IL27 should be assessed in further detail, why can't IFNab suppress here and vice versa, why did IL27 not stand in for IFNab in the other paper? Both should be able to activate STAT1 and therefore should be redundant. The question of whether one or the other is upstream or downstream apparently has been addressed up to a certain point as stated by the authors in the discussion, I think this aspect is important for the novelty of this m/s and should be further explored experimentally.

2. Overall, most symbols in the figures are too small, it is very difficult to distinguish treatment groups.

3. Fig.1A: I do not understand the histogram: % of what are we looking at? Of total CD45+ cells? If yes, then the precise gating strategy should be explained and ideally shown in a supplementary figure. Since CD4 T cells are presumably many more, how does the number of IL-17+ gd T cells compare to that of IL-17+ CD4 T cells? Is that what we see here?

4. Fig.1F: What do these histologies tell me? Please show a pair of micrographs that has similar exposure and brightness.

5. Fig.5: The experiment mixing DCs and gd T cells of different genotypes should be also done with lung, not spleen gd T cells as shown here.

6. Fig. 7C,D are incomprehensible: is this an aSTAT1 ChIP followed by IL17 promoter PCR? The text is wrong I think as it says in presence or absence of IL17, should read IL27? I would like to see revised version that allows me to actually follow the experiment. The M+M section on Chip is too short, as it stands this experiment cannot be evaluated.

7. Another typo when explaining Suppl.Fig.5? In the presence of IL27 or IL-17? Not comprehensible in the present form.

Materials and Methods:

8. The cell purification protocol for epithelial cells is incomprehensible, has to be rewritten: what Ficoll layers, what centrifugations, which fraction was taken etc etc?

9. The main text mentions i.t. addition of S.p. while M+M described a different method, Influenza inoculation is described by 2 different methods and it is not clear when the authors use which type of treatment. What is the real protocol?

Minor concerns:
Typos, some of which turn the meaning of the statements upside down:

Page 8 of the m/s: aIL17 treated mice: survival rate LOWER, not higher, correct???
P1: significantly DECREASED mortality in IL27R-/- mice vs wt mice
P14: deficient, misspelled throughout
P18: wrong syntax

1st Revision - authors' response 26 August 2013

Referee #1 (Comments on Novelty/Model System):

The studies appear to be appropriately done and analyzed in an appropriate mouse model, with verification of important effects in human samples.

Thank you very much for your favorite comments.

Referee #1 (Remarks):

This manuscript by Cao et al presents a thorough and well-crafted investigation of the role of IL-27 and IL-17 on post-flu susceptibility to S. pneumo pneumonia. Their findings suggest that IL-27 induction after flu results in decreased gdT cell-derived IL-17 in response to S. pneumo that mediates decreased neutrophil-mediated protection. Neutralization experiments in mice and verification of IL-27 induction and effects in human samples provide translational relevance. My enthusiasm for this manuscript is high, but I feel it could be further strengthened as noted in my comments below:

Thank you very much for your favorite and valuable comments.

1. The results suggest flu may act on a variety of cell types to induce IL-27, which then has JAK2/STAT1-dependent suppression of IL-17 in gamma delta T cells. A missing link here is how flu is inducing IL-27, especially in the context of the recognized immunosuppressive effects of flu-induced type I IFN. Is induction of IL-27 triggered by type I IFN? This could be addressed by using IFNAR KO mice/cells. The authors mention in the discussion unshown data that uses anti-IFNa Ab, but given the numerous species of type I IFN, this seems an inadequate approach.

Thank you very much for your kind suggestions. To investigate if influenza-induced IL-27 production is regulated by type I IFNs, we firstly studied the relative kinetics of IFN-β versus IL-27 transcript expression (Figure 4D). Detectable levels of IFN-β appeared 1 h after influenza infection and peak at ~3 h, while induction of IL-27p28 or EBI3 appeared delayed relative to IFN-β, with significant mRNA detection occurring 3 h after influenza infection and peaking at ~6h. Furthermore, IFNAR-deficient mice/cells were used according to your kind suggestion. We found that IL-27 production was significantly reduced in IFNAR-deficient BMDC, monocytes, LEC and lymphocytes after influenza infection with or without HkSp co-stimulation when compared with WT cells (Figure 4E). *In vivo*, we also demonstrated that IFNAR-deficient mice after influenza infection had much less IL-27 protein in the lungs when compared with WT mice (Figure 4F). All these data suggest that influenza infection up-regulated IL-27 production in an IFNAR-dependent manner. We have added these new data in the revised manuscript.

2. The authors cite a recent manuscript by Li et al. that implicated gdT cell IL-17 suppression by type I IFN. The authors should more explicitly put their work in context of this. In particular, they should investigate/discuss whether any STAT-1 inducer suppresses IL-17 and whether the effects of type I IFN and IL-27 on gdT cell IL-17 are independent of each other.

Thank you very much for your kind suggestions. In fact, when we prepared this manuscript, Li et al demonstrated that influenza-infected IFNAR-deficient mice were resistant to secondary pneumococcal pneumonia related with increased IL-17A production in γδ T cells (Li et al, 2012, J Virol), but the underlying mechanisms remained undefined. Given that IL-27 production was markedly decreased in IFNAR-deficient mice after influenza infection (Figure 4), we predicted that decreased IL-27 may be the significant mediator of enhanced clearance of secondary pneumococcal pneumonia observed in IFNAR-deficient mice, and determined whether IL-27 treatment *in vivo* could reverse the tolerance to secondary pneumococcal pneumonia in IFNAR-deficient mice. We found that inoculation of exogenous IL-27 could significantly down-regulate IL-17A production (Figure 6A) and the percentage of IL-17A-producing γδ T cells (Figure 6B) in the lungs of IFNAR-deficient mice after secondary pneumococcal infection. Accordingly, IL-27 treatment also significantly decreased neutrophil recruitment (Figure 6C) and MPO activity (Figure 6D) in IFNAR-deficient mice compared with control mice. In addition, IFNAR-deficient mice treated with IL-27 showed significantly higher pulmonary pneumococcal burdens (Figure 6E) and lower survival rates (Figure 6F). The pneumococcal counts and survival rates in IL-27-treated IFNAR-deficient mice were strikingly close to those of virus/*S.pn*-infected WT mice, indicating that the inhibitory effects of Type I IFNs on IL-17A production by γδ T cells to promote postinfluenza pneumococcal pneumonia was mediated via IL-27. However, treatment of IL-27 did not change pneumococcal counts and survival rates in WT mice, suggesting that IL-27 was expressed at a functionally adequate level in WT mice during secondary pneumococcal pneumonia.

Having observed that influenza induced IL-27 production in an IFNAR-dependent manner (Figure 4), and the inhibitory effects of Type I IFNs on IL-17A production by γδ T cells to promote postinfluenza pneumococcal pneumonia were mediated via IL-27 *in vivo* (Figure 6), we further determined whether IL-27 was responsible for the inhibitory effects of IFN-β *in vitro.* Our results showed that supernatants from IFN-β–treated splenocytes inhibited the development of IL-17A-producing γδ T cells stimulated by HkSp and IL-23. When anti–IL-27 antibodies were added to block the IL-27 activity in the supernatants from IFN-β–treated splenocytes, the IFN-β–mediated inhibitory effect on IL-17A production was reversed, as demonstrated by intracellular IL-17 staining and IL-17 secretion analysis from γδ T cells (Figure 8A, 8B). On the other hand, IL-27 suppressed the development of IL-17A-producing γδ T cells from IFNAR-deficient mice (Figure 8C, 8D), indicating that IL-27 activity on γδ T cells was not dependent on IFNAR signalling.

Since early expression of type I IFNs is a molecular signature of influenza virus infection (Katze et al, 2002, Nat Rev Immunol; Shinohara et al, 2008, Immunity), which regulates IL-27 production upon influenza infection as observed in this study, we can conclude that IL-27 signalling occurs downstream of type I IFNs in the suppression of IL-17A production by γδ T cells, thereby promoting secondary pneumococcal infection. We have added these new data in the revised manuscript according to your kind suggestions.

3. Much important data has been put in supplemental materials. If at all possible, some of this should be put in the main figures. For example, the human data in Fig S5 and the establishment of the role of IL-17 in Fig S1. If needed due to space limitation, some of the 'negative' data, for example on non-IL17 cytokines in Fig 2 etc., as well as the 27R expression data in Fig 5, could be

moved to supplemental instead. On a similar note, Fig 2F showing the CFU and mortality data in the context of flu should be moved to the beginning of this figure to establish their model--and then this can be followed by the cytokine analysis etc.

Thank you very much for your kind suggestions. We have put your suggested supplemental materials into the main figures. We also have moved CFU and mortality data in the context of flu to the beginning of Figure 3, and then followed by the cytokine analysis etc.

4. The flow cytometry evaluation of gamma delta T cells should be presented in more detail as supplemental info. For example, in Fig. 1, plots of gamma delta vs. CD45 or total cells should be shown to provide confidence that gating is being done appropriately. Documentation of purity of sorted cells with flow plots should be provided.

Thank you very much. According to your kind suggestions, gating strategy for analysis of IL-17A-producing cells in the lungs of *S. pneumoniae*-infected mice was shown in Supplemental Figure 1. The lymphocytes were initially selected based on forward scatter (FSC) and side scatter (SSC), followed by CD45, CD3, CD4, CD8, γδ T CR and NK1.1 gating, and finally separated into different subsets. Documentation of purity of sorted cells with flow plots was also provides in Supplemental Figure 6: spleens or lungs isolated from mice were sorted by flow cytometry into purified γδ TCR-positive cells. A representative sort was shown for the purity of sorted spleen γδ T cells, which was ~99%.

5. The CFU difference between 27R wt and ko in Fig 3D-E seems very modest (less than a log difference), yet it results in dramatic survival differences. Is this expected, or are other IL-27-mediated host factors contributing to the mortality effects? The correlation between small CFU differences and mortality should be discussed in the context of the authors' experience with this system or by citing appropriate literature. Also, statistics are not provided for survival curves, e.g. Fig 3D-E.

Thank you very much for your kind suggestions. To answer your questions, we also determined pulmonary bacterial burdens as early as 24 h after secondary infection with *S. pneumoniae* and bacteremia rates in the infected-mice. We found that lungs from virus/*S.pn*-infected IL-27R-deficient mice contained over 10-fold fewer median bacterial loads when compared with WT mice as early as 24 h after secondary infection with *S. pneumoniae*, with significant differences persisting for up to 48 h (Figure 4G). Similarly, markedly lower rates of bacteremia were noted in virus/*S.pn*–infected IL-27R-deficient mice when compared with WT mice (Figure 4H). These differences in pulmonary and systemic pneumococcal loads were associated with significantly decreased mortality in IL-27R-deficient mice (Figure 4I)， and this correlation between pneumococcal burdens and mortality was expected during pneumococcal infection, which is consistent with the reports as described in our previous studies and others when studying the protection of novel pneumococcal protein vaccines against pneumococcal infection (Min et al, 2012, Vaccine; Gong et al, 2011, Infect Immun; Giefing et al, 2008, J Exp Med). Hence, the induction of IL-27 didn't affect the viral or pneumococcal clearance in naive mice, but markedly increased susceptibility to secondary pneumococcal infection. We have added these new data and discussed this correlation in the revised manuscript. Besides, statistics for survival curves are also provided in the revised manuscript.

6. In Fig 4/S4, there is nice use of adoptive transfer experiments and recombinant cytokines to establish the role of cell subtypes and cytokines. Use of neutralizing antibody against IL-17 in 27R KO mice would solidify the role of IL-17 in mediating the effects of IL-27.

Thank you very much for your kind suggestions. According to your suggestions, we used neutralizing antibodies against IL-17A in 27R-deficient mice to further solidify the role of IL-17A in mediating the effects of IL-27. IL-17A depletion resulted in a significant decrease of neutrophil recruitment (Supplemental Figure 7A) and MPO activity (Supplemental Figure 7B) in the lungs of IL-27R-deficient mice following secondary *S. pneumoniae* challenge. Besides, the pneumococcal counts in IL-17A-depleted IL-27R-deficient mice were significantly increased (Supplemental Figure 7C), and a significantly enhanced morality was observed in these mice (Supplemental Figure 7D), while isotypical IgG-treated IL-27R-deficient mice had similar parameters with WT mice.

Interestingly, neutralization of IL-17A did not significantly change pulmonary pneumococcal burdens in WT mice (Supplemental Figure 7C), indicating that IL-17A was produced at a functionally ineffective level in WT mice during secondary pneumococcal pneumonia. All together, these data support a mechanism by which influenza induced IL-27, which through inhibition of IL-17A production attenuated neutrophil recruitment and activity in the lung, thereby leading to impaired antibacterial defence and increased susceptibility to secondary infection. We have added these new data in the revised manuscript.

7. In Fig 5D-E, the DC-gdT coculture system nicely addresses the role of these cell subsets. Including conditions where both subsets are 27R-deficient would clarify whether this would account for the full effect of 27R deficiency on IL-17. If not, the potential reasons should be discussed. In addition, 27R-deficiency in gdT cells almost fully accounted for the 17 suppression by flow but only partially by ELISA--how do the authors account for this? Finally, the authors should clarify if the 17 data reflects gating on gdT cells or not.

Thank you very much for your kind suggestions. To answer your questions, we performed experiments using BMDC, lung γδ T cells or both from IL-27R-deficient mice. We found that IL-27 was dependent IL-27R expression on lung γδ T cells to exert its suppressive effects, whereas its presence on BMDC was partially required, because the expansion of IL-17A-secreting lung γδ T cells was partially but not completely suppressed when co-culturing lung γδ T cells deficient in IL-27R and WT DC, while IL-27 had no inhibitory effects on the expansion of IL-17A-secreting IL-27R-deficient lung γδ T cells when co-culturing with IL-27R-deficient BMDC (Figure 7C). Further ELISA assays also showed that IL-17A protein secretion was partially decreased in co-culture of IL-27R-deficient lung γδ T cells and WT DC but strongly inhibited in co-culture of WT lung γδ T cells and IL-27R-deficient DC by IL-27 (Figure 7D). We further repeated our experiments using BMDC, spleen γδ T cells or both from IL-27R-deficient mice, and also found that IL-27 was dependent on IL-27R expression on spleen γδ T cells to inhibit IL-17A production, while its expression on DC was partially required (Supplemental Figure 10A, 10B). Therefore, although there was somewhat variation between intracellular IL-17A in gated γδ T cells by flow cytometry and secreted IL-17A in culture supernatants by ELISA from 3 independent experiments (each was performed with cells isolated from 3 mice), our data clearly suggest that the suppressed production of IL-17A in γδ T cells induced by IL-27 was regulated by IL-27R on γδ T cells dominantly and at least in part via the expression of IL-27R on DC. Besides, we have clarified that γδ T cells in co-culture with BMDC were gated for intracellular IL-17A staining assay in the revised manuscript according to your kind suggestions.

8. Fig 7: specific activity of the Jak inhibitor should be shown/referenced. If possible, the effect of STAT3 deficiency in comparison to STAT1 deficiency should be shown. Why isn't mortality shown for the adoptive transfer experiment?

Thank you very much for your kind suggestions. Specific activity of the Jak inhibitor has been referenced (Young et al, 2012, J Immunol) and determined by Western blot (Figure 9A). At the concentration of JI-1 (1 nM) that selectively inhibited Jak2 and Tyk2, IL-27 failed to inhibit IL-17A production by γδ T cells in co-culture with BMDC stimulated by HkSp (Figure 9B), demonstrating that suppression of IL-17A in γδ T cells by IL-27 was mediated by Jak2/Tyk2 activity. Since STAT1/STAT3 can be directly phosphorylated by Jak2/Tyk2 and mediates several biological functions of IL-27 (Hunter & Kastelein, 2012, Immunity), we then used specific inhibitors for STAT1 (fludarabine) and STAT3 (S31-201) (Mir et al, 2012, J Biol Chem) to inhibit the activity of STAT1 and STAT3, respectively. At the concentration of fludarabine (50 µM) and S31-201 (10 µM) that selectively inhibited STAT1 and STAT3, respectively (Figure 9C), we found that STAT1 inhibitor fludarabine could reverse the inhibitory effects of IL-27 on the development of IL-17A-producing γδ T cells (Figure 9D). In the presence of STAT3 inhibitor S31-201, IL-27 could efficiently suppress IL-17A production by γδ T cells, which is consistent with a recent report that STAT3 was dispensable for the development of IL-17–producing γδ T cells (Shibata et al, 2011, Blood). According to your kind suggestions, we have shown the effect of STAT3 deficiency in comparison to STAT1 deficiency in the revised manuscript. In the subsequent work, we thus examined whether the suppressive effects of IL-27 on IL-17A production in γδ T cells to promote

secondary pneumococcal pneumonia after influenza infection was dependent on STAT1 using STAT1-deficient mice.
In addition, we have demonstrated that mice receiving STAT1-deficient γδ T cells had a significantly lower mortality when compared with that of mice receiving WT γδ T cells (Figure 9L) according to your suggestions.

9. Better explanation/rationale for each set of experiments would be helpful--for example, rationale for looking at IL-27 in Fig 3, explaining that gp130 and WSX-1 are 27 receptors in Fig 5.

Thank you very much for your kind suggestions. In the revised Figure 4, since IL-27 is an important regulatory cytokine that can limit ongoing immune responses depending on context (Hunter & Kastelein, 2012), we first examined the induction of IL-27 *in vivo*.
Functional IL-27 receptor comprises a heterodimer consisting of WSX-1 and gp130 (Hunter & Kastelein, 2012, Immunity; Villarino et al, 2003, J Immunol). To examine the direct effects of IL-27 on IL-17A production by γδ T cells *in vitro*, the surface expression of IL-27 heterodimer receptor on FACS-purified spleen γδ T cells or BMDC was firstly characterized by flow cytometry in the revised supplemental Figure 9.
As described above, we have provided better explanation/rationale for these experiments in the revised manuscript according to your kind suggestions. .

10. For Fig 2C and similar graphs, I would suggest sticking with the convention and putting the independent variable (IL17) on the y axis instead of the x axis--this would also make it consistent with the rest of the graphs.

Thank you very much. We have putted the independent variable (IL17) on the y axis instead of the x axis in the revised manuscript according to your kind suggestions.

11. Better explanation of ELISA on lung homogenates in the methods is needed (how much material was tested, how was it done).

Thank you very much. For lung homogenates, whole lungs were removed, taking care to dissect away lymph nodes. The lungs were homogenized in 1 ml of PBS supplemented with protease inhibitor cocktail (Roche Applied Science), followed by centrifugation at 1000 x g for 10 min. Supernatants were stored at -80°C until further use. Cytokine/chemokine/growth factor in total lung lysates was measured by ELISA according to the manufacturer's protocol. We have provided this explanation of ELISA on lung homogenates in the revised manuscript according to your kind suggestions.

Referee #2 (Remarks):

Cao et al show here that influenza infection leads to high lung IL-27 levels which are able to block IL-17 induction in gd T cells, leading to lower neutrophil counts, higher bacterial load and higher susceptibility upon secondary bacterial infection. This process is STAT1 dependent and also active in human gd T cells. Overall, this study is well performed and controlled, with amazingly clear data. Many of the concerns as listed below refer to clarity of expression, which sometimes lacks to the point of rendering comprehension impossible.

Thank you very much for your valuable comments.

Major concerns:

1. General concern: There is similar, published data (which the authors also quote) showing that IFNab suppresses IL17 from gd T cells - so this m/s appears to delineate a parallel mechanism. The relationship between IFNab and IL27 should be assessed in further detail, why can't IFNab

suppress here and vice versa, why did IL27 not stand in for IFNab in the other paper? Both should be able to activate STAT1 and therefore should be redundant. The question of whether one or the other is upstream or downstream apparently has been addressed up to a certain point as stated by the authors in the discussion, I think this aspect is important for the novelty of this m/s and should be further explored experimentally.

Thank you very much for your kind suggestions. To answer your questions, we further used IFNAR-deficient mice to assess the relationship between type I IFNs and IL-27. We firstly studied the relative kinetics of IFN-β versus IL-27 transcript expression (Figure 4D). Detectable levels of IFN-β appeared 1 h after influenza infection and peak at ~3 h, while induction of IL-27p28 or EBI3 appeared delayed relative to IFN-β, with significant mRNA detection occurring ~3 h after influenza infection and peaking at ~6h. Remarkably, IL-27 production was significantly reduced in IFNAR-deficient BMDC, monocytes, LEC and lymphocytes after influenza infection with or without HkSp co-stimulation compared with WT cells (Figure 4E). *In vivo*, we also found that IFNAR-deficient mice after influenza infection had much less IL-27 protein in the lungs when compared with WT mice (Figure 4F). All these data suggest that influenza infection up-regulated IL-27 production in an IFNAR-dependent manner.

In fact, when we prepared this manuscript, Li et al demonstrated that influenza-infected IFNAR-deficient mice were resistant to secondary pneumococcal pneumonia related with increased IL-17A production in γδ T cells (Li et al, 2012, J Virol), but the underlying mechanisms remained undefined. Given that IL-27 production was markedly decreased in IFNAR-deficient mice after influenza infection (Figure 4), we predicted that decreased IL-27 may be the significant mediator of enhanced clearance of secondary pneumococcal pneumonia observed in IFNAR-deficient mice, and determined whether IL-27 treatment *in vivo* could reverse the tolerance to secondary pneumococcal pneumonia in IFNAR-deficient mice. We found that inoculation of exogenous IL-27 could significantly down-regulate IL-17A production (Figure 6A) and the percentage of IL-17A-producing γδ T cells (Figure 6B) in the lungs of IFNAR-deficient mice after secondary pneumococcal infection. Accordingly, IL-27 treatment also significantly decreased neutrophil recruitment (Figure 6C) and MPO activity (Figure 6D) in IFNAR-deficient mice compared with control mice. In addition, IFNAR-deficient mice treated with IL-27 showed significantly higher pulmonary pneumococcal burdens (Figure 6E) and lower survival rates (Figure 6F). The pneumococcal counts and survival rates in IL-27-treated IFNAR-deficient mice were strikingly close to those of virus/*S.pn*-infected WT mice, indicating that the inhibitory effects of Type I IFNs on IL-17A production by γδ T cells to promote postinfluenza pneumococcal pneumonia was mediated via IL-27. However, treatment of IL-27 did not change pneumococcal counts and survival rates in WT mice, suggesting that IL-27 was expressed at a functionally adequate level in WT mice during secondary pneumococcal pneumonia.

Evidence in support of that IL-27 mediated the inhibitory effects of IFNAR signalling on IL-17A production by γδ T cells during secondary pneumococcal infection was further provided by our demonstration that neutralization of IL-27 abrogated the inhibitor effects of IFN-β on the development of IL-17A-pruducing γδ T cells *in vitro* (Figure 8A, 8B). On the other hand, IL-27 suppressed the development of IL-17A-producing γδ T cells from IFNAR-deficient mice (Figure 8C, 8D), indicating that IL-27 activity on γδ T cells was not dependent on IFNAR signalling.

Since early expression of type I IFNs is a molecular signature of influenza virus infection (Katze et al, 2002, Nat Rev Immunol; Shinohara et al, 2008, Immunity), which regulates IL-27 production upon influenza infection as observed in this study, we can conclude that IL-27 signalling occurs downstream of type I IFNs in the suppression of IL-17A production by γδ T cells, thereby promoting secondary pneumococcal infection. We have added these new data in the revised manuscript according to your kind suggestions.

2. Overall, most symbols in the figures are too small, it is very difficult to distinguish treatment groups.

Thank you very much for your suggestions. We have enlarged the figures or using different colour figures in the revised manuscript.

3. Fig.1A: I do not understand the histogram: % of what are we looking at? Of total CD45+ cells? If yes, then the precise gating strategy should be explained and ideally shown in a supplementary figure. Since CD4 T cells are presumably many more, how does the number of IL-17+ gd T cells

compare to that of IL-17+ CD4 T cells? Is that what we see here?

Thank you very much for your kind suggestions. In the Figure 2 of revised manuscript, histograms showed the percentages of various CD45+ lung cell populations (total CD45+ cells) positive for intracellular IL-17A. The gating strategy for analysis of IL-17A-producing cells in the lungs of *S. pneumoniae*-infected mice was shown in supplemental Figure 1 according to your kind suggestions. The lymphocytes were initially selected based on forward scatter (FSC) and side scatter (SSC), followed by CD45, CD3, CD4, CD8, γδ T CR and NK1.1 gating, and finally separated into different subsets.
Since CD4+ T cells are presumably many more, we agree that it is not precise to compare the percentages of IL-17A-producing γδ T cells and IL-17A-producing CD4 + T cells. Therefore, comparison of absolute number of IL-17A-producing γδ T cells and IL-17A-producing CD4+ T cells during pneumococcal infection was shown in Figure 2C of revised manuscript, and we found that the IL-17A response in γδ T cells was both faster and stronger than CD4+ T cells during pneumococcal infection. We have added these new data in the revised manuscript according to your kind suggestions.

4. Fig.1F: What do these histologies tell me? Please show a pair of micrographs that has similar exposure and brightness.

Thank you very much. A pair of micrographs that has similar exposure and brightness was shown in Figure 2F of revised manuscript, which demonstrated that WT mice had the infiltration of large amounts of inflammatory cells, especially neutrophils, while γδ T cell–deficient mice had less immune cell infiltration.

5. Fig.5: The experiment mixing DCs and gd T cells of different genotypes should be also done with lung, not spleen gd T cells as shown here.

Thank you very much. According to your kind suggestions, both lung and spleen γδ T cells were used in the experiment of mixing DCs and γδ T cells of different genotypes in the revised manuscript (Figure 7C, 7D, and supplemental Figure 10A, 10B). We found that IL-27 was dependent on IL-27R expression on spleen γδ T cells to inhibit IL-17A production, while its expression on DC was partially required.

6. Fig. 7C,D are incomprehensible: is this an aSTAT1 ChIP followed by IL17 promoter PCR? The text is wrong I think as it says in presence or absence of IL17, should read IL27? I would like to see revised version that allows me to actually follow the experiment. The M+M section on Chip is too short, as it stands this experiment cannot be evaluated.

Thank you very much. We have corrected this error in the revised manuscript. It was in the presence or absence of IL-27. Besides, we have rewritten the CHIP assay section in the revised manuscript. Briefly, DNA-bound transcription factors in treated-γδ T cells were cross-linked by infusing complete medium containing 1% formaldehyde for 10 min followed by sonication of cell lysates to shear DNA. After preclearing with protein A agarose beads (Upstate), cell lysates were immunoprecipitated with specific anti-STAT1 antibodies (Cell Signalling) or rabbit control IgG (Santa Cruz Biotechnology) overnight at 4°C. After washing and elution, crosslinks were reversed at 65°C for 4 h. The eluted DNA was then purified and analysed by either relative-PCR or quantitative-PCR with IL-17A promoter site-specific primers. The binding to STAT1 sites in the IL-17A promoter region was performed using the following primers: GGA GAG ATG GCT CAG CAG TTA AG; reverse primer, TGG TTT CTG GGA ATT GAA CTC A. The Ct value of each sample was normalized to the corresponding input value and expressed as fold induction relative to the normal rabbit serum control, which was calculated as 1.0. We have added these new words in the revised manuscript according to your kind suggestions.

© EMBO

7. Another typo when explaining Suppl.Fig.5? In the presence of IL27 or IL-17? Not comprehensible in the present form.

Thank you very much. We have corrected this error in the revised manuscript. It was in the presence or absence of IL-27.

Materials and Methods:

8. The cell purification protocol for epithelial cells is incomprehensible, has to be rewritten: what Ficoll layers, what centrifugations, which fraction was taken etc. etc.?

Thank you very much for your kind suggestions. We have rewritten the cell purification protocol for murine epithelial cells in the revised manuscript as followings: Murine lung epithelial cells (LEC) were isolated as described previously (You et al 2002, Am J Physiol Lung Cell Mol Physiol; Kim et al, 2011, J Immunol). Briefly, lungs were perfused with 20 ml of sterile PBS via the right ventricle until they were visually free of blood, and then filled (2 ml per lung) via the airway with RPMI 1640 with 2.5% FBS (HyClone Laboratories), 80 U elastase, and 0.05 mg/ml trypsin (Sigma-Aldrich). After incubation at 37˚C for 25 min, the lungs were homogenized. Then the homogenate was centrifuged at 2000 rpm for 2 min, and the supernatant fraction containing the cell suspension was layered on top of an isotonic Percoll solution (1.082 g/ml, GE Healthcare, Little Chalfont, U.K.) and centrifuged for 25 min at 1500 rpm at 4˚C. The cells at the interface between Percoll layers were removed and cultured in plates coated with anti-Fc receptor mAbs (BD Pharmingen) at 37˚C for 30 min. Then the nonadherent cells were collected, centrifuged, and resuspended in DMEM containing with 10% FBS and 1% penicillin-streptomycin. Finally, these resuspended cells were cultured to >90% confluence in DMEM containing with 10% FBS for future experiments. The purity of these isolated LEC was identified using anti-mouse pan-cytokeratin mAbs (Abcam) and anti-mouse FcgIII/II receptor mAbs (BD Pharmingen). Flow cytometric analysis showed the purity of LEC was >90%.

9. The main text mentions i.t. addition of S.p. while M+M described a different method, Influenza inoculation is described by 2 different methods and it is not clear when the authors use which type of treatment. What is the real protocol?

Thank you very much for your kind comments. We have clarified this confusion in the revised manuscript. For influenza infection, mice were infected using an Inhalation Exposure System (Glass-Col, USA) for influenza virus infection, a dose of 200 PFU of influenza A PR/8/34 H1N1 (in 40 µl sterile PBS) from a frozen stock or control PBS was given. For *S.pneumoniae* infection, mice were anesthetized with pentobarbital sodium intraperitoneally (i.p.) (30mg/kg weight), and then 5,000 CFU *S.pneumoniae* in 30ul sterile PBS was administered intranasally into mice as described in our previous studies (Gong et al, 2011, Infect Immun), which mimicked the natural route of pneumococcal infection

Minor concerns:

Typos, some of which turn the meaning of the statements upside down:
Page 8 of the m/s: aIL17 treated mice: survival rate LOWER, not higher, correct???
P1: significantly DECREASED mortality in IL27R-/- mice vs. wt mice
P14: deficient, misspelled throughout
P18: wrong syntax

Thank you very much for your kindness. We have corrected these minor errors in the revised manuscript.

2nd Editorial Decision 06 September 2013

Thank you for the submission of your revised manuscript to EMBO Molecular Medicine. We have now received the enclosed reports from the referees that were asked to re-assess it. As you will see the reviewers are now supportive and I am pleased to inform you that we will be able to accept your manuscript pending the following final amendments:

-Please follow the advice of referee 1 and rewrite your article in places in order to shorten it, avoiding redundancy and improving readability.
-Both referees also suggest correcting figure 2D. In addition, referee 1 suggests moving some figure panels to the SI. As the article contains 10 figures, we would kindly encourage you to do so.
-Ethics: please provide an ethical statement for the obtention and use of the human samples.

Please submit your revised manuscript within two weeks. I look forward to seeing a revised form of your manuscript as soon as possible.

***** Reviewer's comments *****

Referee #1 (Comments on Novelty/Model System):

Thorough and detailed investigation of phenomenon and mechanisms with appropriate controls and verification with human samples.

Referee #1 (Remarks):

The authors have addressed my previous concerns. I feel they have substantially strengthened the manuscript, in particular with data placing the effects of IL27 downstream of IFNAR and strengthening the data on the role of STAT1. The current manuscript is logically organized, but it is heavily laden with data (a good thing!) that may make length and readability an editorial concern. It could likely benefit from editing for succinctness, and perhaps the discussion could be shortened to minimize repetition of the results. The following are minor points that could be addressed at the final discretion of the authors and editors:

1. If needed for space, Figure 2 (or at least major portions of it) could be moved to supplemental to document that IL-17 is mainly coming from gdT cells. Similarly, Fig 4C and E showing the in vitro production of IL-27 from different cellular subsets could be made supplemental and still have this figure convey the major findings regarding the role of IL-27.

2. It would be conventional to present all the CFU data on a log scale.

3. Fig 2D seems to have mislabeled y-axes--I assume it should be similar to Fig 2E.

Referee #2 (Remarks):

authors have addressed adequately the referees' concerns. fig. 2d has wrong annotations, this has to be fixed before publication.

2nd Revision - authors' response 19 September 2013

Referee #1 (Comments on Novelty/Model System):

Thorough and detailed investigation of phenomenon and mechanisms with appropriate controls and verification with human samples.

Thank you very much for your kind comments.

Referee #1 (Remarks):

The authors have addressed my previous concerns. I feel they have substantially strengthened the manuscript, in particular with data placing the effects of IL27 downstream of IFNAR and strengthening the data on the role of STAT1. The current manuscript is logically organized, but it is heavily laden with data (a good thing!) that may make length and readability an editorial concern. It could likely benefit from editing for succinctness, and perhaps the discussion could be shortened to minimize repetition of the results. The following are minor points that could be addressed at the final discretion of the authors and editors:

Thank you very much for your favourite comments. We have edited and shortened the discussion section in the revised manuscript according to your kind suggestions.

1. *If needed for space, Figure 2 (or at least major portions of it) could be moved to supplemental to document that IL-17 is mainly coming from gdT cells. Similarly, Fig 4C and E showing the in vitro production of IL-27 from different cellular subsets could be made supplemental and still have this figure convey the major findings regarding the role of IL-27.*

Thank you very much for your kind suggestions. We have moved Figure 2, Figure 4C and E to the supplemental information according to your kind suggestions in the revised manuscript.

2. *It would be conventional to present all the CFU data on a log scale.*

Thank you very much. We have presented all the CFU data on a log scale according to your kind suggestions in the revised manuscript.

3. *Fig 2D seems to have mislabelled y-axes--I assume it should be similar to Fig 2E.*

Thank you very much. We have corrected this error in the revised manuscript according to your kind suggestions.

Referee #2 (Remarks):

authors have addressed adequately the referees' concerns. fig. 2d has wrong annotations, this has to be fixed before publication.

Thank you very much. We have corrected this error in the revised manuscript according to your kind suggestions.
