# Supplementary material for: Activation of IL-27 signalling promotes development of postinfluenza pneumococcal pneumonia
Source: EMBO Mol Med. 2013 Oct 29;6(1):120–40. doi: 10.1002/emmm.201302890 (PMC3936494; doi:10.1002/emmm.201302890)
Supplement: Supplementary file 2 [file emmm0006-0120-sd2.pdf]

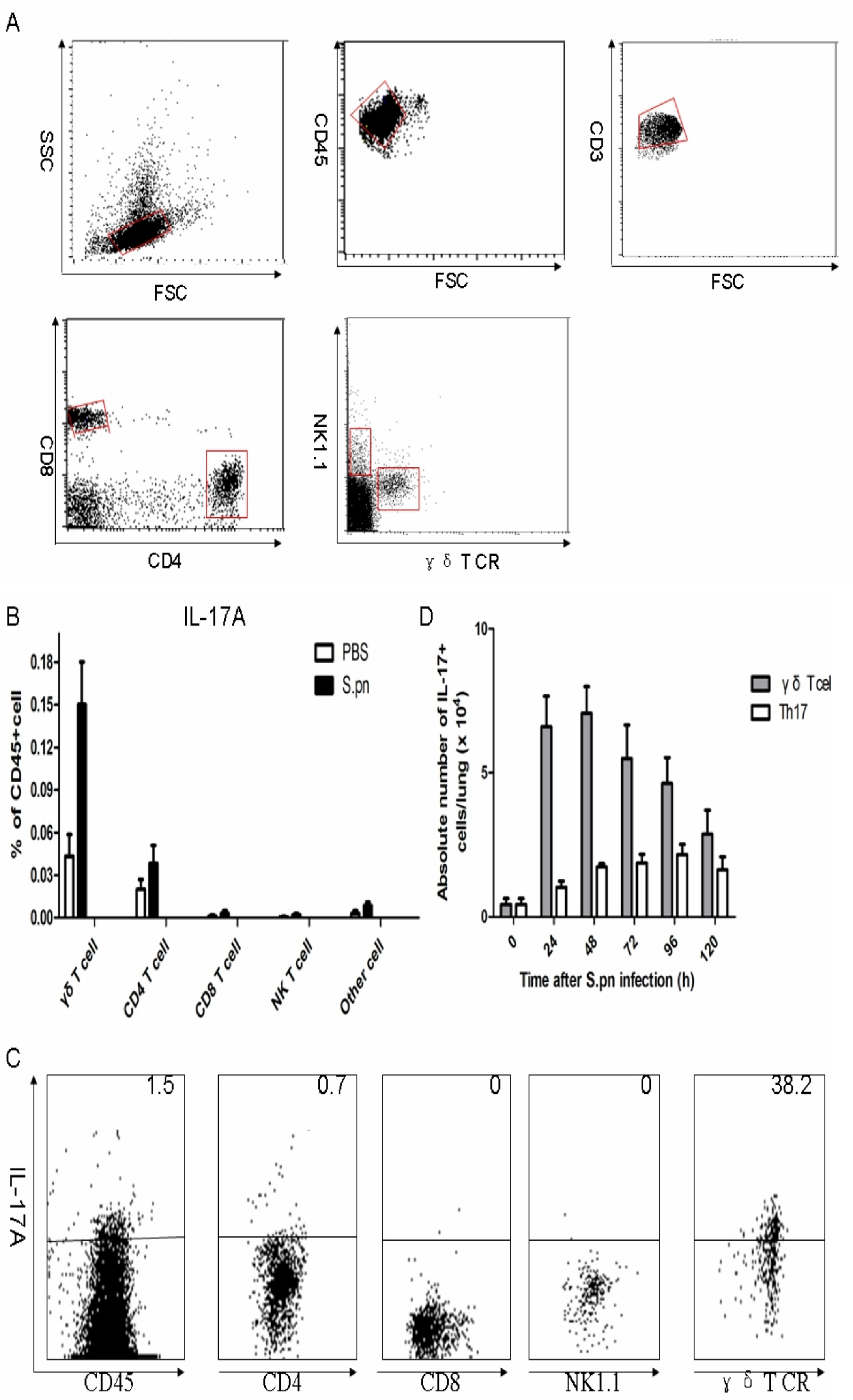
A
SSC
FSC
CD45
FSC
CD3
FSC
CD8
CD4
NK1.1
γ δ TCR
B
IL-17A
PBS
S.pn
% of CD45+cell
0.18
0.15
0.12
0.09
0.06
0.03
0.00
γδ T cell
CD4 T cell
CD8 T cell
NK T cell
Other cell
D
Absolute number of IL-17+ cells/lung (x 10^4)
10
5
0
γ δ Tcel
Th17
0
24
48
72
96
120
Time after S.pn infection (h)
C
IL-17A
1.5
0.7
0
0
38.2
CD45
CD4
CD8
NK1.1
γ δ TCR

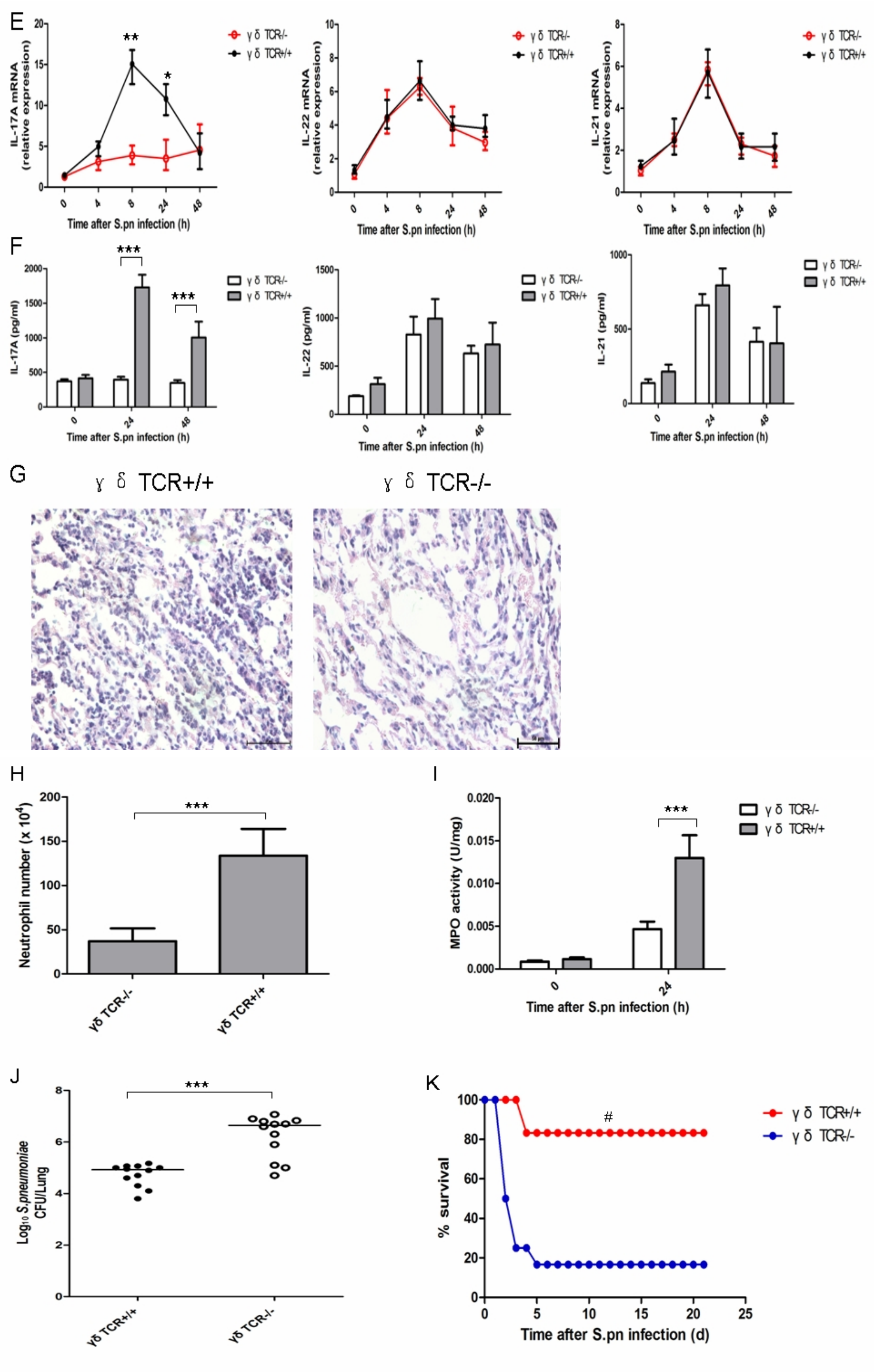

Supplemental Figure 1: γδ T cells were the dominant producers of IL-17A during pneumococcal pneumonia. (**A**) Gating strategy for analysis of IL-17A-producing cells

in the lungs of *S. pneumoniae*-infected mice. The lymphocytes were initially selected based on forward scatter (FSC) and side scatter (SSC), followed by CD45, CD3, CD4, CD8, NK1.1 and γδ T CR gating, and finally separated into different subsets. (**B**) Histograms showed the percentages of various CD45+ lung cell populations positive for intracellular IL-17A (n=5). Lung cells were isolated from C57BL/6 mice 24 h after intranasal infection with *S. pneumoniae*. Cells were cultured for 6 h with 500 ng/ml PdBU and 500 ng/ml ionomycin in the presence of 5 μg/ml brefeldin A, and intracellular staining assay for IL-17A was performed by flow cytometry. (**C**) Intracellular IL-17A in γδ T, CD4, CD8, and NKT cells at 24 h after intranasal infection with *S. pneumoniae*. (**D**) Comparison of absolute number of IL-17A–producing γδ T cells and CD4+ T cells in the lungs isolated at various time points after *S.pneumoniae* challenge (n=5). (**E**) Cytokine mRNA levels in the lungs from γδ T cell–deficient and WT mice performed at 0, 4, 8, and 24 h after pneumococcal challenge (n=5). (**F**) Cytokine protein levels in the lungs from γδ T cell–deficient and WT mice at 24 h following pneumococcal infection (n=5). (**G**) Representative photomicrographs of lung sections at 24 h after pneumococcal challenge from γδ T cell–deficient and WT mice labeled with H&E stain. (**H**) Lung neutrophil numbers in γδ T cell–deficient and WT mice at 24 h after pneumococcal infection (n=3). (**I**) Lung MPO activity in γδ T cell–deficient and WT mice (n=5). (**J**) Pulmonary pneumococcal burdens at 48 h in γδ T cell–deficient and WT mice after pneumococcal challenge (n=12). (**K**) Survival for γδ T cell–deficient and WT mice after pneumococcal challenge (n=12). *p<0.05, **p<0.01, ***p<0.001 when compared between γδ T cell–deficient and WT mice; #p<0.05 when compared with γδ T cell–deficient mice.
