# Supplementary material for: Activation of IL-27 signalling promotes development of postinfluenza pneumococcal pneumonia
Source: EMBO Mol Med. 2013 Oct 29;6(1):120–40. doi: 10.1002/emmm.201302890 (PMC3936494; doi:10.1002/emmm.201302890)
Supplement: Supplementary file 3 [file emmm0006-0120-sd3.pdf]

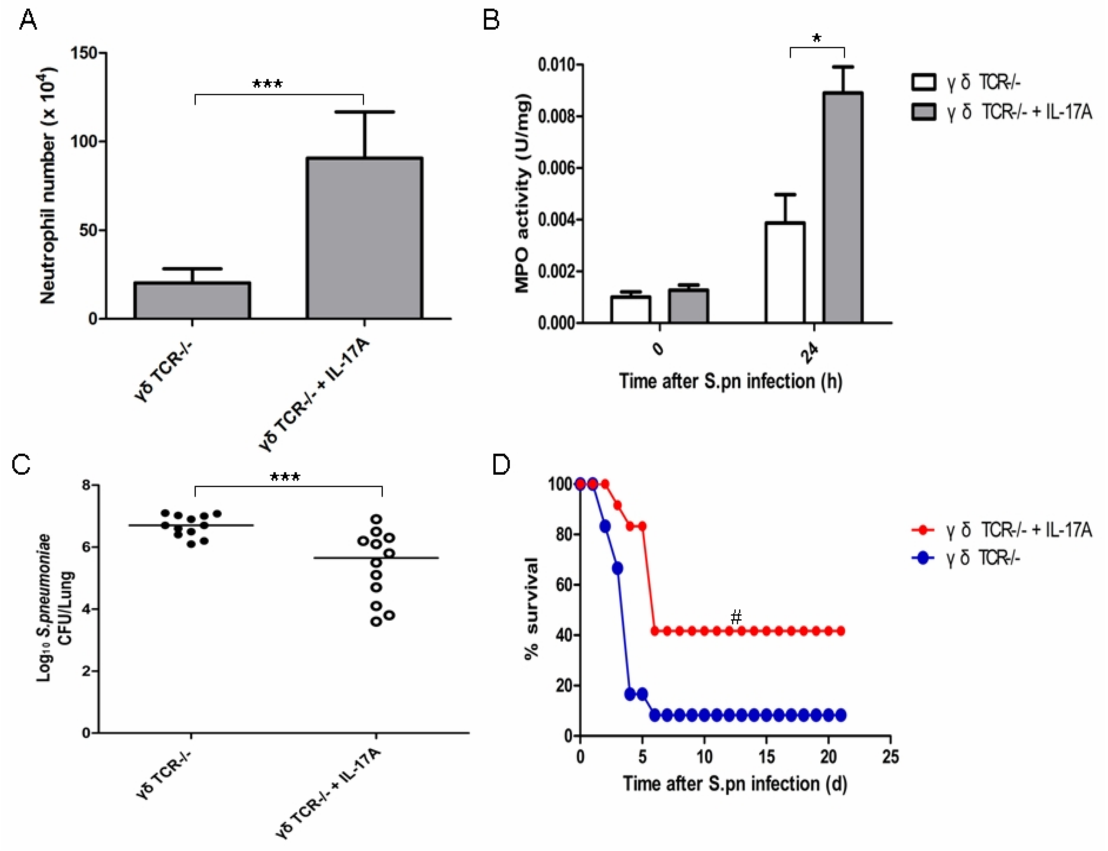

Supplemental Figure 2: Administration of recombinant IL-17A enhanced the protection against pneumococcal infection in γδ T cell–deficient mice. Recombinant murine IL-17A (10 μg) was given i.t. into WT mice followed by intranasal pneumococcal challenge. (**A**) Lung neutrophil numbers at 24 h in γδ T cell–deficient mice treated with IL-17A protein or PBS control after pneumococcal challenge (n=5). (**B**) Lung MPO activity at 0 and 24 h in γδ T cell–deficient mice treated with IL-17A protein or PBS control (n=5). (**C**) Pulmonary pneumococcal burdens at 48 h in γδ T cell–deficient mice treated with IL-17A protein or PBS control (n=12). (**D**) Survival for γδ T cell–deficient mice treated with IL-17A protein or PBS control after pneumococcal challenge (n=12). *$p<0.05$, ***$p<0.001$ when compared between groups denoted by horizontal lines. #$p<0.05$ when compared with mice treated with PBS control.
