# Supplementary material for: Activation of IL-27 signalling promotes development of postinfluenza pneumococcal pneumonia
Source: EMBO Mol Med. 2013 Oct 29;6(1):120–40. doi: 10.1002/emmm.201302890 (PMC3936494; doi:10.1002/emmm.201302890)
Supplement: Supplementary file 4 [file emmm0006-0120-sd4.pdf]

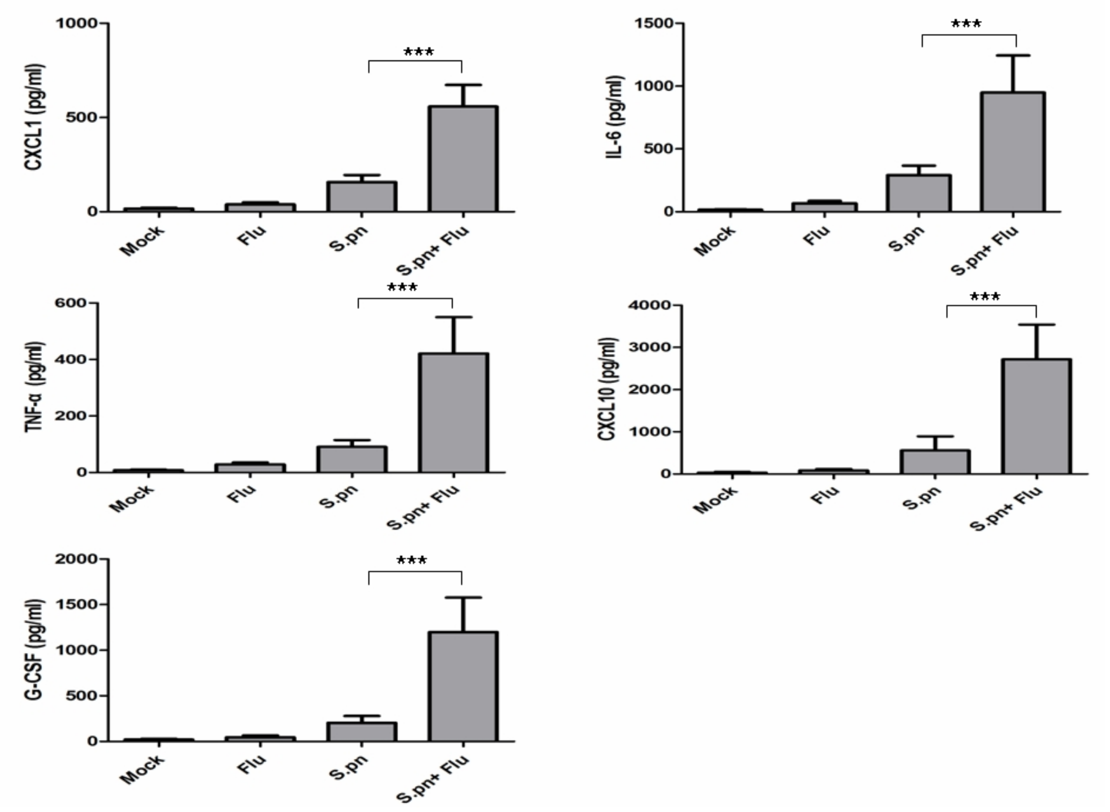

Supplemental Figure 3: Cytokine/chemokine/growth factor levels in the lungs of mice with influenza infection, *S.pneumoniae* infection or secondary pneumococcal infection following primary influenza infection. Lung homogenates were obtained and analyzed at 24 h after secondary challenge with *S. pneumoniae* (n=5). ****p*<0.001 when compared between groups denoted by horizontal lines.
