# Supplementary material for: Activation of IL-27 signalling promotes development of postinfluenza pneumococcal pneumonia
Source: EMBO Mol Med. 2013 Oct 29;6(1):120–40. doi: 10.1002/emmm.201302890 (PMC3936494; doi:10.1002/emmm.201302890)
Supplement: Supplementary file 5 [file emmm0006-0120-sd5.pdf]

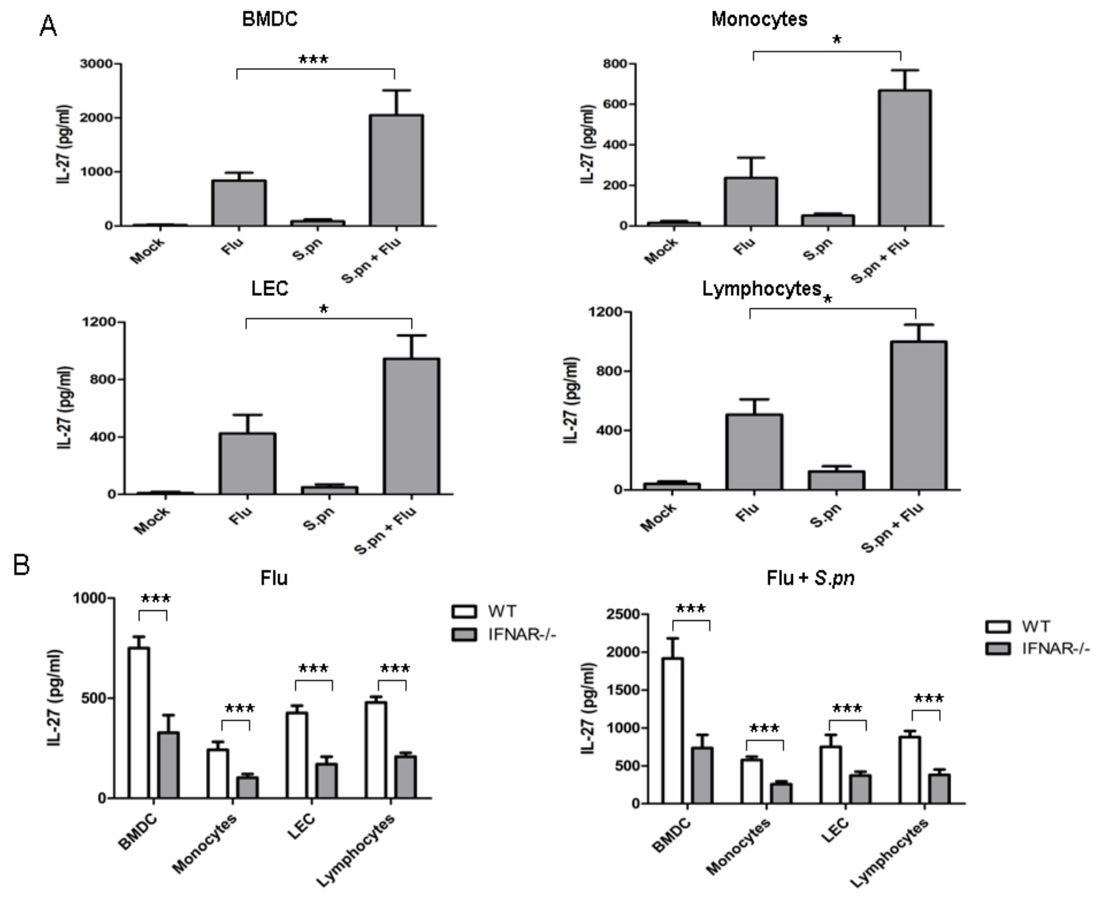

Supplemental Figure 4: IL-27 production in different murine cell types after infection with influenza virus and *S.pneumoniae*. (**A**) IL-27 produced by murine BMDC, monocytes, LEC and lymphocytes infected with influenza virus at multiplicity of infection (MOI) =1 or HkSp (1 x 10^8 CFU/ml). After 24 h, ELISA was performed to measure the IL-27 concentrations in the culture supernatants (n=3). (**B**) IL-27 production in BMDC, monocytes, LEC and lymphocytes isolated from IFNAR-deficient or WT mice after infection with influenza virus (MOI=1) in the presence or absence of HkSp (1 x 10^8 CFU/ml) *in vitro* (n=3). **p*<0.05, ****p*<0.001 when compared between groups denoted by horizontal lines
