# Supplementary material for: Activation of IL-27 signalling promotes development of postinfluenza pneumococcal pneumonia
Source: EMBO Mol Med. 2013 Oct 29;6(1):120–40. doi: 10.1002/emmm.201302890 (PMC3936494; doi:10.1002/emmm.201302890)
Supplement: Supplementary file 6 [file emmm0006-0120-sd6.pdf]

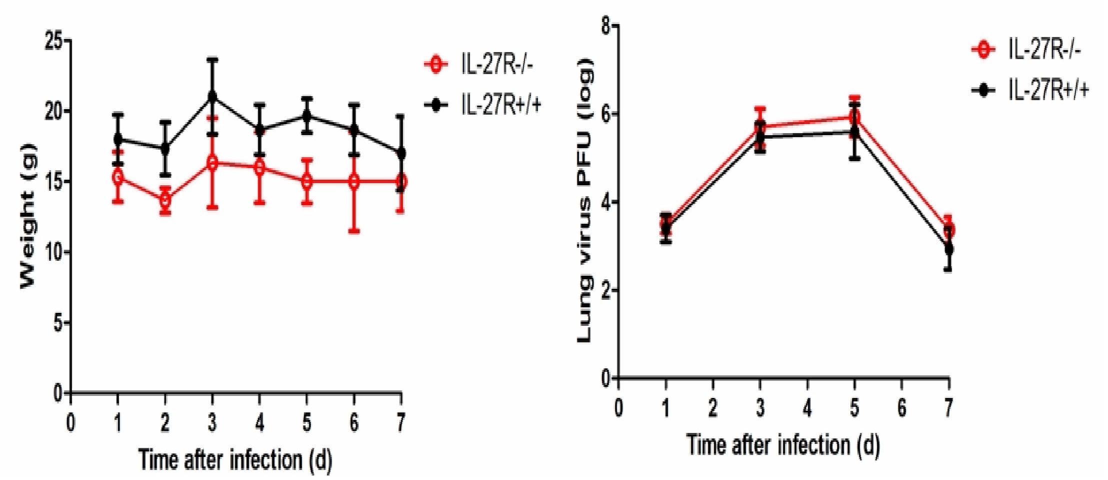

Supplemental Figure 5: IL-27R-deficient mice were similarly sensitive as WT mice upon influenza virus infection. IL-27R-deficient and WT mice were infected with 200 PFUs of influenza virus. Weights were obtained in mice following influenza virus infection, and the mice were sacrificed at indicated times for assessment of viral PFUs in lung homogenates (n=5).
