# Supplementary material for: Activation of IL-27 signalling promotes development of postinfluenza pneumococcal pneumonia
Source: EMBO Mol Med. 2013 Oct 29;6(1):120–40. doi: 10.1002/emmm.201302890 (PMC3936494; doi:10.1002/emmm.201302890)
Supplement: Supplementary file 7 [file emmm0006-0120-sd7.pdf]

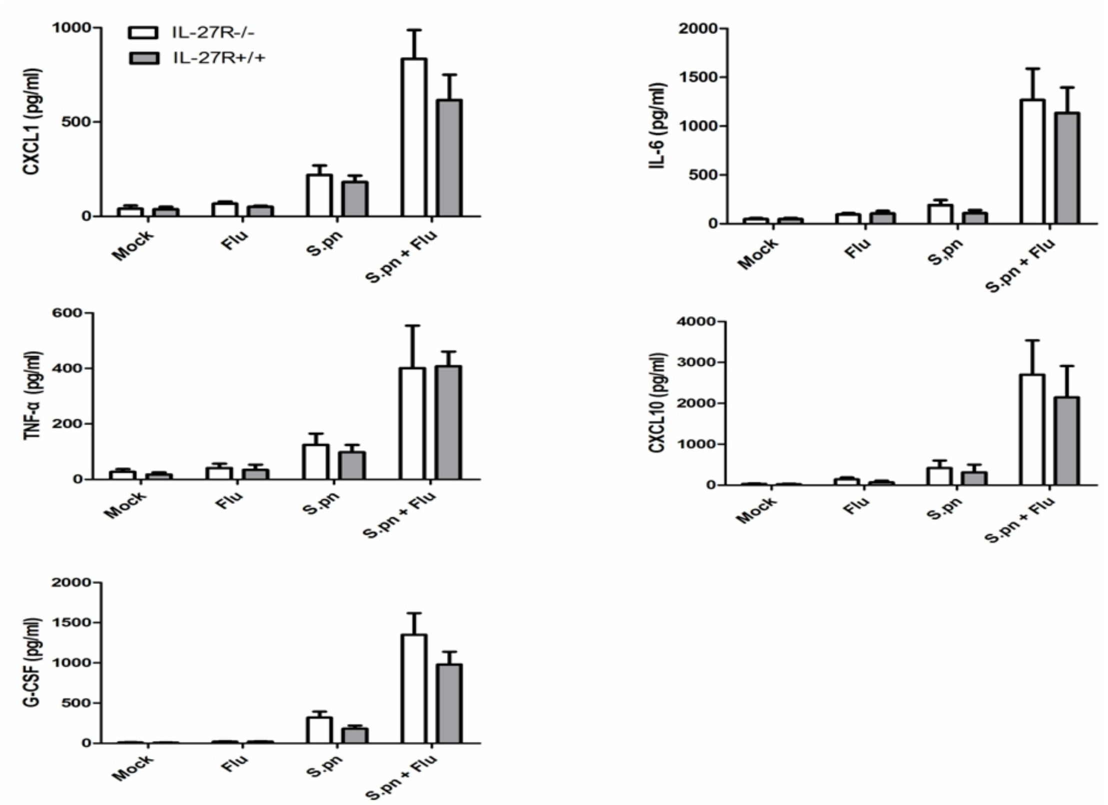

Supplemental Figure 6: Cytokine/chemokine/growth factor levels in the lungs from IL-27R–deficient and WT mice at 24 h after influenza infection alone, *S.pneumoniae* infection alone or secondary pneumococcal infection following primary influenza infection (n=5).
