# Supplementary material for: Activation of IL-27 signalling promotes development of postinfluenza pneumococcal pneumonia
Source: EMBO Mol Med. 2013 Oct 29;6(1):120–40. doi: 10.1002/emmm.201302890 (PMC3936494; doi:10.1002/emmm.201302890)
Supplement: Supplementary file 8 [file emmm0006-0120-sd8.pdf]

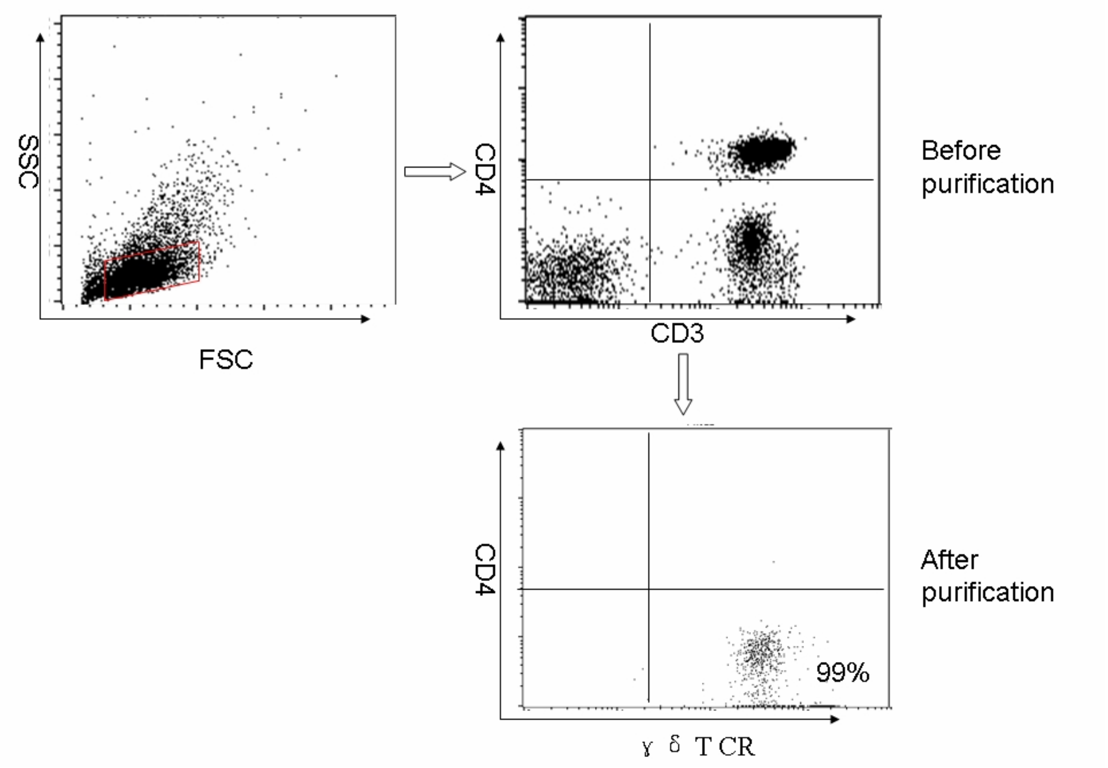

Supplemental Figure 7: Spleens or lungs isolated from mice were sorted by flow cytometry into purified γδ TCR-positive cells. A representative sort was shown for the purity of sorted spleen γδ T cells, which was ~99%.
