# Supplementary material for: Activation of IL-27 signalling promotes development of postinfluenza pneumococcal pneumonia
Source: EMBO Mol Med. 2013 Oct 29;6(1):120–40. doi: 10.1002/emmm.201302890 (PMC3936494; doi:10.1002/emmm.201302890)
Supplement: Supplementary file 9 [file emmm0006-0120-sd9.pdf]

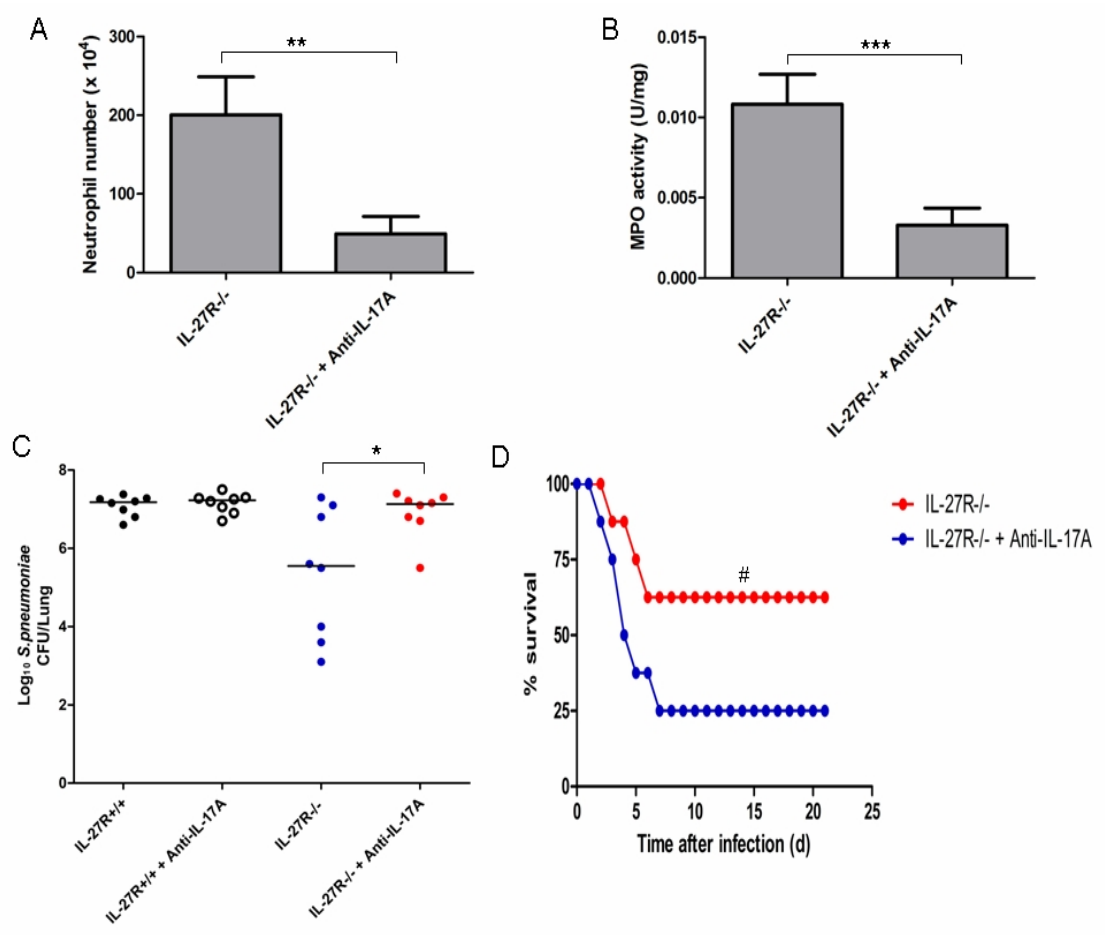

Supplemental Figure 8: IL-17A played a key role in the clearance of secondary pneumococcal infection in IL-27R-deficient mice. IL-27R-deficient or WT mice were infected with influenza virus, followed 5 days later by intranasal pneumococcal challenge. Anti-17A neutralizing antibodies (1.5 mg) or control IgG were administered i.p. 24 h prior to pneumococcal challenge. (**A**) Lung neutrophil numbers at 24 h in IL-27R-deficient mice treated with anti-IL-17A antibodies or control IgG after secondary pneumococcal challenge (n=5). (**B**) Lung MPO activity at 24 h in IL-27R-deficient mice treated with anti-IL-17A antibodies or control IgG (n=5). (**C**) Pulmonary pneumococcal burdens at 48 h in IL-27R-deficient or WT mice treated with anti-IL-17A antibodies or control IgG after secondary pneumococcal challenge (n=12). (**D**) Survival for IL-27R-deficient mice treated anti-IL-17A antibodies or control IgG after secondary pneumococcal challenge (n=12). *$p<0.05$, **$p<0.01$, ***$p<0.001$ when compared between groups denoted by horizontal lines. #$p<0.05$ when compared with mice treated with anti-IL-17A antibodies.
