# Supplementary material for: Activation of IL-27 signalling promotes development of postinfluenza pneumococcal pneumonia
Source: EMBO Mol Med. 2013 Oct 29;6(1):120–40. doi: 10.1002/emmm.201302890 (PMC3936494; doi:10.1002/emmm.201302890)
Supplement: Supplementary file 10 [file emmm0006-0120-sd10.pdf]

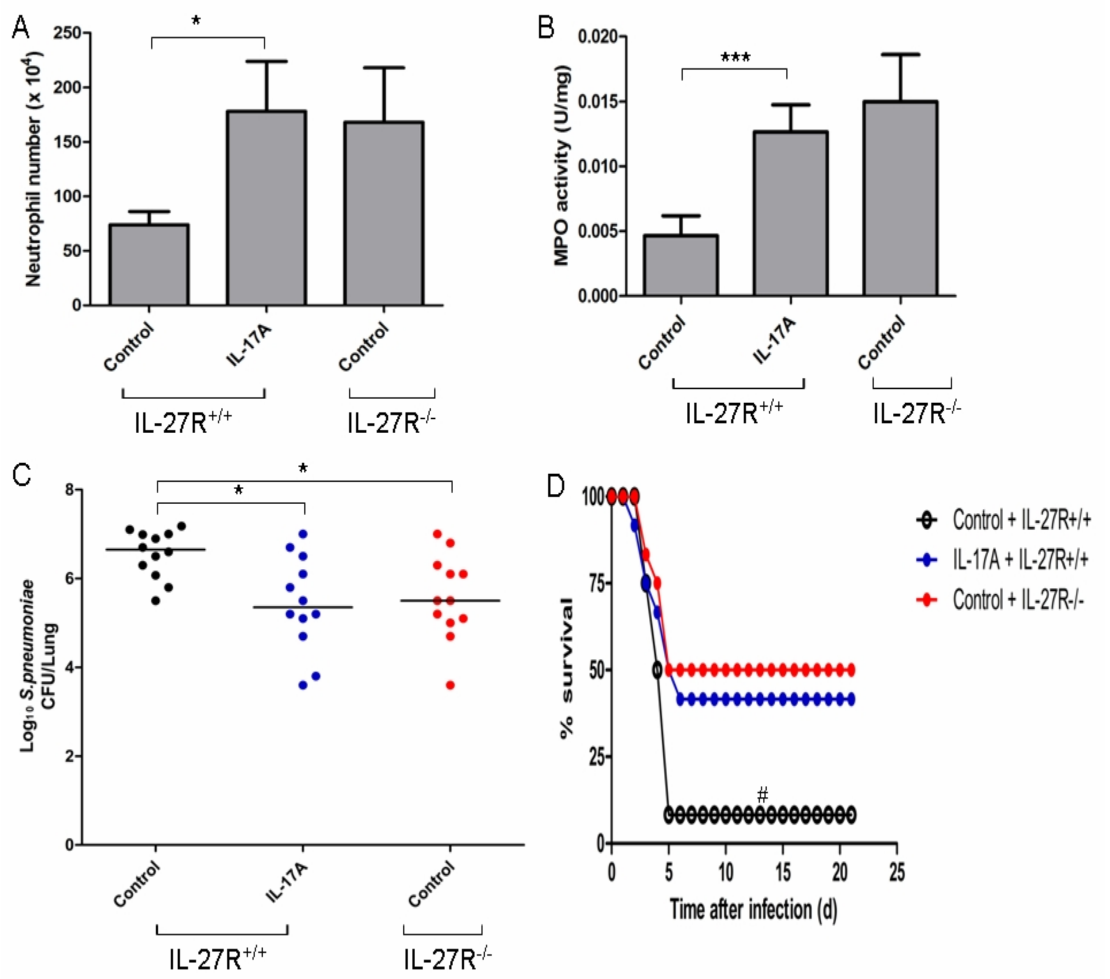

Supplemental Figure 9: Exogenous IL-17A administration rescued influenza-infected WT mice challenged secondarily with *S.pneumoniae*. Recombinant murine IL-17A protein was given i.t. into mice followed by intranasal pneumococcal challenge. IL-27-deficient mice treated with PBS served for comparison. (**A**) Lung neutrophil numbers at 24 h in WT mice treated with IL-17A protein or PBS control following secondary pneumococcal challenge (n=5). (**B**) Lung MPO activity at 24 h in WT mice treated with IL-17A protein or PBS control following secondary pneumococcal challenge (n=5). (**C**) Pulmonary pneumococcal burdens at 48 h in WT mice treated with IL-17A protein or PBS control (n=12). (**D**) Survival for WT mice treated with IL-17A protein or PBS control following secondary pneumococcal challenge (n=12). **p*<0.05, ****p*<0.001 when compared between groups denoted by horizontal lines. #*p*<0.05 when compared with mice treated with IL-17A protein.
