# Supplementary material for: Activation of IL-27 signalling promotes development of postinfluenza pneumococcal pneumonia
Source: EMBO Mol Med. 2013 Oct 29;6(1):120–40. doi: 10.1002/emmm.201302890 (PMC3936494; doi:10.1002/emmm.201302890)
Supplement: Supplementary file 11 [file emmm0006-0120-sd11.pdf]

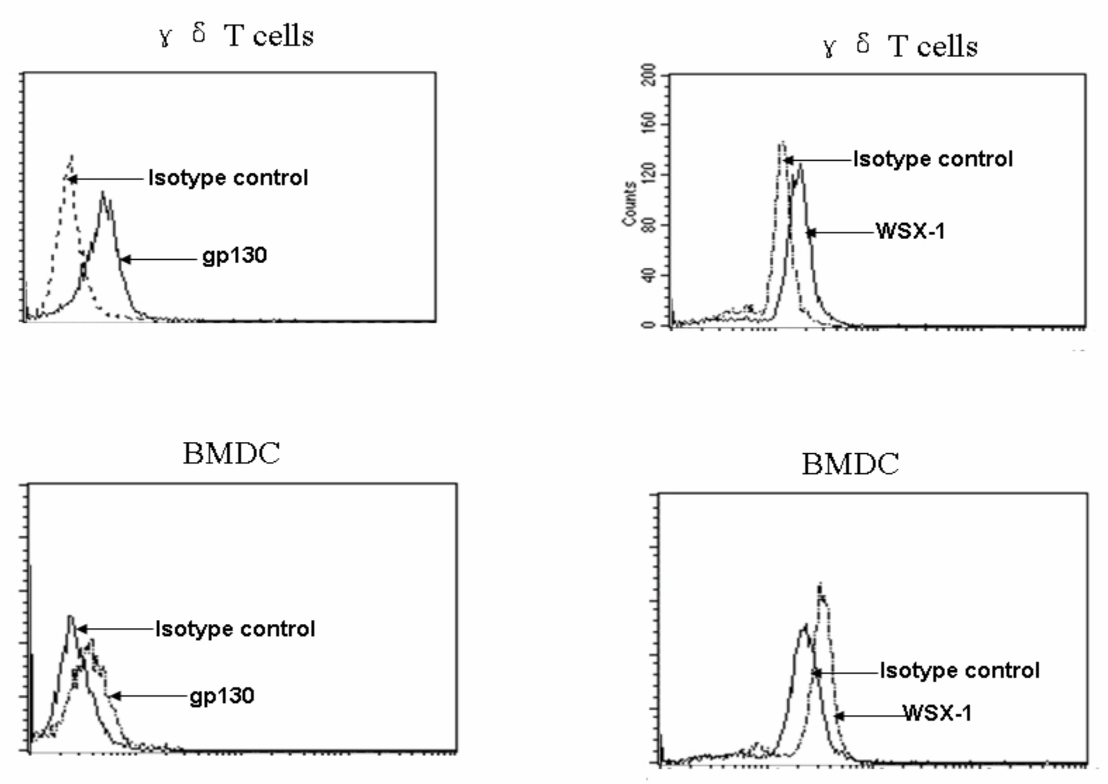

Supplemental Figure 10: The expression of gp130 and WSX-1 chains was evaluated by flow cytometry on highly purified spleen γδ T cells and BMDC. A representative example illustrated the expression of gp130 and WSX-1 on both cells from 3 independent experiments.
