# Supplementary material for: Activation of IL-27 signalling promotes development of postinfluenza pneumococcal pneumonia
Source: EMBO Mol Med. 2013 Oct 29;6(1):120–40. doi: 10.1002/emmm.201302890 (PMC3936494; doi:10.1002/emmm.201302890)
Supplement: Supplementary file 12 [file emmm0006-0120-sd12.pdf]

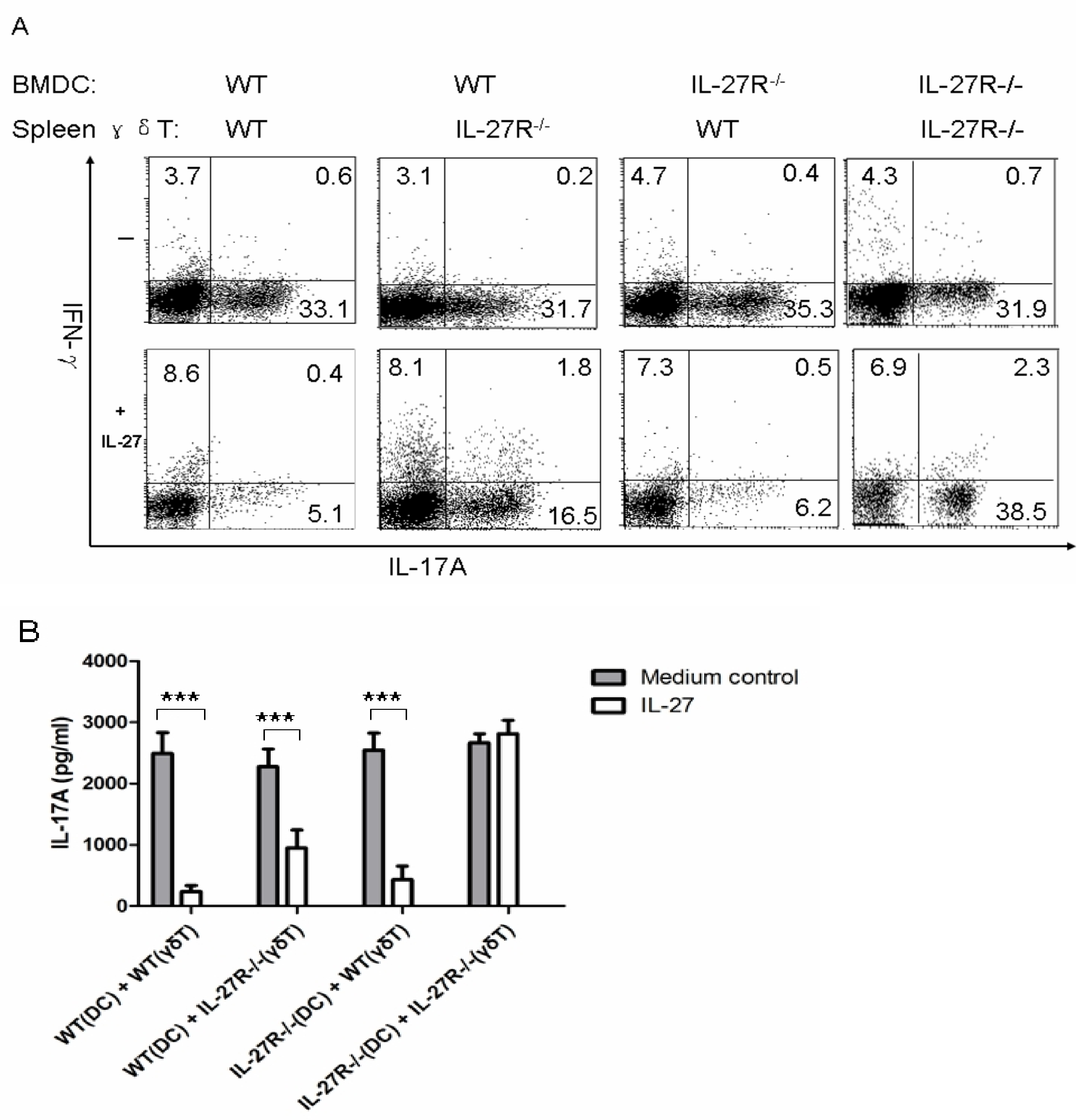

Supplemental Figure 11: IL-27 inhibited IL-17A production by spleen γδ T cells *in vitro*. (**A**) FACS analysis of IL-17A and IFN-γ expression in FACS-sorted spleen γδ T cells from IL-27R-deficient or WT mice, which were co-cultured with BMDC from IL-27R-deficient or WT mice activated by HkSp (1 x 10^8 CFU/ml) in the presence or absence of IL-27 (100 ng/ml) for 72 h. Spleen γδ T cells were gated for FACS analysis. (**B**) IL-17A concentrations in the supernatants of spleen γδ T cells from IL-27R-deficient or WT mice, which were co-cultured with BMDC from IL-27R-deficient or WT mice activated by HkSp in the presence or absence of IL-27 for 72 h. Results were from 3 independent experiments, and each was performed with cells isolated from 3 mice. ***p<0.001 when compared between groups denoted by horizontal lines.
