# Supplementary material for: Activation of IL-27 signalling promotes development of postinfluenza pneumococcal pneumonia
Source: EMBO Mol Med. 2013 Oct 29;6(1):120–40. doi: 10.1002/emmm.201302890 (PMC3936494; doi:10.1002/emmm.201302890)
Supplement: Supplementary file 13 [file emmm0006-0120-sd13.pdf]

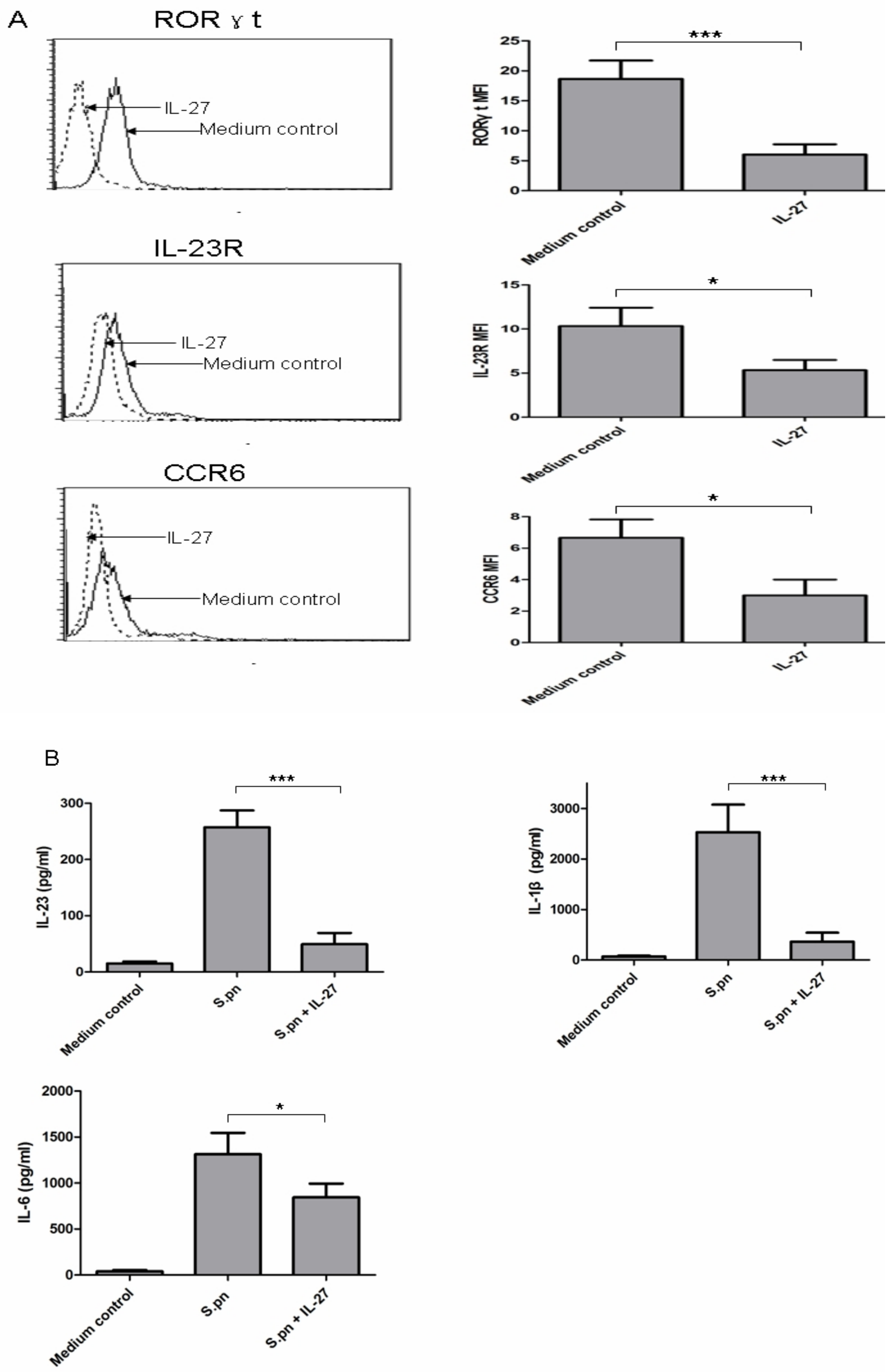

Supplemental Figure 12: IL-27 inhibited RORγt, IL-23R and CCR6 expression in γδ T cells and cytokine production in DC. (**A**) IL-27 down-regulated RORγt, IL-23R and

CCR6 expression in spleen γδ T cells co-cultured with BMDC infected with HkSp (1 x 10^8 CFU/ml) in the presence or absence of IL-27 (100 ng/ml). Intracellular RORγt and surface IL-23R and CCR6 expression by γδ T cells were measured by flow cytometry at 72 h after stimulation. Representative histograms illustrated the expression of RORγt, IL-23R and CCR6 by γδ T cells from 3 independent experiments. (**B**) IL-27 inhibited IL-17A-polarizing cytokines from BMDC. BMDC were stimulated with HkSp (1 x 10^8 CFU/ml) in the presence or absence of IL-27 (100 ng/ml). After 72h, the cell-free culture supernatants were assayed for cytokine levels by ELISA. Results were from 3 independent experiments, and each was performed with cells isolated from 3 mice. *p<0.05, ***p<0.001 when compared between groups denoted by horizontal lines.
