# Supplementary material for: Activation of IL-27 signalling promotes development of postinfluenza pneumococcal pneumonia
Source: EMBO Mol Med. 2013 Oct 29;6(1):120–40. doi: 10.1002/emmm.201302890 (PMC3936494; doi:10.1002/emmm.201302890)
Supplement: Supplementary file 14 [file emmm0006-0120-sd14.pdf]

Table 1. Primer sequences used for real-time PCR

Primer	Sequence
IL-17A	(F) 5’-TCAACCGTTCCACGTCACCCT-3’
	(R)5’-GTCCAGCTTTCCCTCCGCATT-3’
IL-22	(F) 5’-GTGAGAAGCTAACGTCCATC-3’
	(R) 5’-GTCTACCTCTGGTCTCATGG-3’
IL-21	(F) 5’-TCAGCTCCACAAGATGTAAAGGG-3’
	(R) 5’-GGGCCACGAGGTCAATGAT-3’
IL-27p28	(F) 5'-CTCTGCTTCCTCGCTACCAC-3'
	(R) 5'-GGGGCAGCTTCTTTTCTTCT-3'
EBI3	(F) 5'- ACCCATTGAAGCCACGACTT -3'
	(R) 5'- AGTATTGCATCCAGGTGTCAGCT -3'
IFN-β	(F) 5'- AGCTCCAAGAAAGGACGAACAT-3'
	(R) 5'- GCCCTGTAGGTGAGGTTGATCT-3'
GAPDH	(F) 5'- CACTACCGTACCTGACACCA-3'
	(R) 3'- TCCACCACCCTGTTGCTGTA-5'

F: forward; R: reverse
